# Impaired calcium signaling in astrocytes modulates autism spectrum disorder-like behaviors in mice

**DOI:** 10.1038/s41467-021-23843-0

**Published:** 2021-05-31

**Authors:** Qian Wang, Ying Kong, Ding-Yu Wu, Ji-Hong Liu, Wei Jie, Qiang-Long You, Lang Huang, Jian Hu, Huai-De Chu, Feng Gao, Neng-Yuan Hu, Zhou-Cai Luo, Xiao-Wen Li, Shu-Ji Li, Zhao-Fa Wu, Yu-Long Li, Jian-Ming Yang, Tian-Ming Gao

**Affiliations:** 1 State Key Laboratory of Organ Failure Research, Key Laboratory of Mental Health of the Ministry of Education, Guangdong-Hong Kong-Macao Greater Bay Area Center for Brain Science and Brain-Inspired Intelligence, Guangdong Province Key Laboratory of Psychiatric Disorders, Department of Neurobiology, School of Basic Medical Sciences, Southern Medical University, Guangzhou, P. R. China; 2 The Ministry of Education of China, School of Basic Medical Sciences, Institute of Neuroscience and Department of Neurology of the Second Affiliated Hospital of Guangzhou Medical University, Guangzhou, P. R. China; 3 State Key Laboratory of Membrane Biology, Peking University School of Life Sciences, Beijing, China; 4 PKU-IDG/McGovern Institute for Brain Research, Beijing, China; 5 Peking-Tsinghua Center for Life Sciences, Academy for Advanced Interdisciplinary Studies, Peking University, Beijing, China; 6 Chinese Institute for Brain Research, Beijing, China

**Keywords:** Astrocyte, Autism spectrum disorders

## Abstract

Autism spectrum disorder (ASD) is a common neurodevelopmental disorder. The mechanisms underlying ASD are unclear. Astrocyte alterations are noted in ASD patients and animal models. However, whether astrocyte dysfunction is causal or consequential to ASD-like phenotypes in mice is unresolved. Type 2 inositol 1,4,5-trisphosphate 6 receptors (IP3R2)-mediated Ca^2+^ release from intracellular Ca^2+^ stores results in the activation of astrocytes. Mutations of the IP3R2 gene are associated with ASD. Here, we show that both IP3R2-null mutant mice and astrocyte-specific IP3R2 conditional knockout mice display ASD-like behaviors, such as atypical social interaction and repetitive behavior. Furthermore, we show that astrocyte-derived ATP modulates ASD-like behavior through the P2X2 receptors in the prefrontal cortex and possibly through GABAergic synaptic transmission. These findings identify astrocyte-derived ATP as a potential molecular player in the pathophysiology of ASD.

## Abstract (web summary)

Astrocytes contribute to autism spectrum disorder (ASD) pathophysiology. Here, the authors show that IP3R2 conditional KO mice show ASD-like behaviours and identify astrocyte-derived ATP as a modulator of these behaviours in mice.

## Introduction

Autism spectrum disorder (ASD) is a developmental mental disorder characterized by impaired social interaction and communication, and restricted and repetitive interests or activities, and it affects 1 in 160 children globally, according to the World Health Organization (WHO fact sheet on autism 2018); however, the mechanisms underlying ASD remain largely unknown. The role of neurons in the pathogenesis of ASD have been the focus of a large body of research^1–4^. Nevertheless, it has also been shown that astrocytes play an important role in ASD^5^. Notably, in postmortem brain tissue from donors affected by ASD, the altered expression of astrocyte markers, such as glial fibrillary acidic protein (GFAP), S100β, aquaporin-4, connexin 43 and excitatory amino acid transporter 1, has been reported;^6–13^ similar findings have also been reported in animal models of autism^14,15^. Using induced pluripotent stem cells from patients affected by ASD, recent studies have further demonstrated that human induced pluripotent stem cells-derived astrocytes compromise neuronal development; in contrast, control-derived astrocytes rescue the morphological and synaptic defects of ASD neuronal cocultures^16,17^. These findings suggest that astrocytes may be involved in the pathological process of ASD. However, it is unknown whether astrocyte dysfunction represents a cause or a consequence of ASD-like phenotypes in mice.

Astrocytes as the most abundant glial cells in the central nervous system, contribute to many critical brain functions during early development and in adulthood, such as neurogenesis^18^, synaptic development^19^, synaptic transmission and plasticity^20^, and regulate behaviors under both physiological and pathological conditions^21–24^. Astrocytic activation is manifested by an increase in cytoplasmic calcium signals mainly mediated by type 2 inositol 1,4,5-trisphosphate receptors (IP3R2)^21,25–27^, and IP3R2 gene has been identified among the genes affected by rare de novo copy number variants in ASD patients^28–30^. However, whether astrocytic dysfunctions induced by IP3R2 deficits contribute to the pathophysiology of ASD is unclear. In this study, by taking advantage of IP3R2 null and Aldh1l1::Cre^ER^ – IP3R2 floxed animals with impaired astrocytic IP3R2-mediated signaling, we demonstrate that astrocyte-derived ATP is involved in the modulation of ASD-like behaviors in mice.

## Results

Previous studies have shown that IP3R2 knockout (IP3R2 KO) mice (Supplementary Fig. 1a, b) show selective dysfunction of astrocytes but not neurons^21,25,31^. We thus used this mouse line to evaluate the role of astrocytes in the pathophysiology of ASD-like phenotypes. Disturbance of intracellular calcium signals induced by a Gq-linked G protein-coupled receptors (GPCRs) agonist cocktail in astrocytes, but not neurons, from IP3R2 KO mice was confirmed in our previous study^21^. To further verify these findings, we employed astrocyte-specific *GfaABC*_*1*_*D* promoter to express cytosolic GCaMP6m in astrocytes located in the medial prefrontal cortex (mPFC), collected mPFC slices 2 weeks after in vivo virus microinjections, imaged Ca^2+^ signals in GCaMP6m-expressing astrocytes using two-photon microscope, and analyzed and quantified the basic properties of the Ca^2+^ signals by GECIquant (Supplementary Fig. 2a, b). We found that the frequency, amplitude and duration of astrocyte somatic Ca^2+^ signals were markedly decreased in IP3R2 KO mice compared to WT controls (Supplementary Fig. 2c-g). This somatic Ca^2+^ signals could be dramatically enhanced by a GPCRs agonist cocktail (500 μM ATP, 50 μM DHPG,10 μM Carbachol) only in IP3R2 WT mice but not in IP3R2 KO mice (Supplementary Fig. 2h-k). Ca^2+^ signals in astrocytic processes were also measured and quantified under both spontaneous (Supplementary Fig. 2c-g) and GPCRs agonist cocktail-induced (Supplementary Fig. 2l-n) conditions. We found that while the frequency and amplitude (but not the duration) of spontaneous Ca^2+^ signals in astrocytic processes were evidently reduced in IP3R2 KO mice (Supplementary Fig. 2c-g), cocktail-induced Ca^2+^ signals in astrocytic processes were largely spared (Supplementary Fig. 2l-m). These findings are consistent with past work showing that astrocyte somatic Ca^2+^ signals require IP3R2, and Ca^2+^ signals in astrocytic processes are mediated by both IP3R2-dependent and IP3R2-independent mechanisms^27^.

**Fig. 1:**
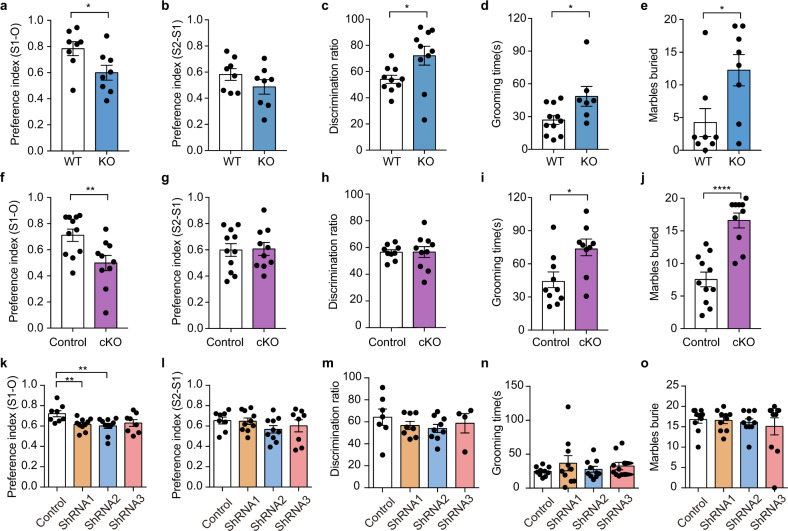
IP3R2 null mutant (IP3R2 KO) and Aldh1L1-CreER:IP3R2^loxp/loxp^ (IP3R2 cKO) mice exhibit ASD-like behaviors. **a**, **b** Impaired social interaction in IP3R2 KO mice in the three-chamber test. **a** Social preference (object versus stranger 1, S1-O, *t*_14_ = 2.353, *P* = 0.0338, *n* = 8). **b** Social novelty recognition (S2-S1, *t*_14_ = 1.341, *P* = 0.2013, *n* = 8). **c** Enhanced recognition memory in IP3R2 KO mice in the novel object recognition test (*U* = 20, *P* = 0.0232, *n* = 10). **d**, **e** Increased repetitive behavior in IP3R2 KO mice in the self-grooming test (**d** *t*_18_ = 2.471, *P* = 0.0251, *n* = 11/7) and the marble-burying test (**e** *t*_14_ = 2.488, *P* = 0.0261, *n* = 8). **f**, **g** Same as (**a**, **b**) but for IP3R2 cKO mice (**f** *t*_19_ = 2.873, *P* = 0.0097, *n* = 11/10; **g** *t*_19_ = 0.9303, *P* = 0.3639, *n* = 11/10). **h** Same as (**c**) but for IP3R2 cKO mice (*U* = 40, *P* = 0.7197, *n* = 9/10). **i**, **j** Same as (**d**, **e**) but for IP3R2 cKO mice (**i** *t*_17_ = 2.845, *P* = 0.0112, *n* = 10/9; **j** *t*_19_ = 5.633, *P* = 0.2 × 10^−4^, *n* = 11/10). **k**, **l** Same as (**a**, **b**) but for IP3R2 knockdown C57BL/6 J mice (**k** Control vs. ShRNA1, *t*_16_ = 0.3078, *p* = 0.0072, *n* = 8/10; Control vs. shRNA2, *t*_16_ = 0.3065, *p* = 0.0074, *n* = 8/10; **l** Control vs. shRNA1, *t*_16_ = 0.1498, *p* = 0.8828, *n* = 8/10; Control vs. shRNA2, *t*_16_ = 1.704, *p* = 0.1076, *n* = 8/10). **m** Same as (**c**) but for IP3R2 knockdown C57BL/6J mice. **n**, **o** Same as (**d**, **e**) but for IP3R2 knockdown C57BL/6J mice. WT, wild-type mice; KO, IP3R2 null mutant mice; control, Aldh1L1-CreER mice; cKO, IP3R2 cKO mice. Data are presented as mean ± SEM; two-tailed unpaired *t* test (**a**, **b**, **d**, **e**, **f**, **g**, **i**, **j**, **k**, **l**, **m**); Mann–Whitney U-test (**c**, **h**, **n**, **o**). *P < 0.05, **P < 0.01, ****P < 0.0001. Each data point represents an individual mouse. Comparisons with no asterisk had a *P* > 0.05 and were considered not significant. Source data are provided as a Source data file.

Aberrant reciprocal social interaction is a core symptom of ASD^32^. To investigate whether the deletion of the IP3R2 gene engenders ASD-related behaviors, we first evaluated social interaction by using the three-chamber assay. We found that IP3R2 KO mice displayed impaired social approach but normal social novelty preference (Fig. 1a, b). During the habituation period, both IP3R2 KO and wild-type mice had no preference for two empty cages (Supplementary Fig. 3a). Once a stranger mouse was introduced into one chamber, wild-type mice spent more time in the mouse-containing chamber than in the empty chamber and socialized more frequently with the stranger mouse. In contrast, IP3R2 KO mice showed no preference for the stranger mouse (Fig. 1a). When a second stranger mouse was introduced into the empty chamber, IP3R2 KO mice showed a similar preference as wild-type mice did for the second stranger mouse (Fig. 1b). The behavioral abnormalities in IP3R2 KO mice did not result from changes in anxiety levels or locomotor activity, as no alterations were observed in the elevated plus-maze (EPM) test (Supplementary Fig. 4a-c) or open field test (OFT) (Supplementary Fig. 4d). Furthermore, IP3R2 KO mice exhibited enhanced recognition memory, as assessed by the novel object recognition (NOR) test (Fig. 1c, Supplementary Fig. 4e-g). Taken together, these results indicate that IP3R2 KO mice display pronounced deficits in social interaction.

ASD is characterized by repetitive behaviors^32^. To test for repetitive behaviors in IP3R2 KO mice, we performed the self-grooming assay and marble-burying test. In the grooming task, we found that IP3R2 KO mice displayed significantly longer self-grooming time than that displayed by wild-type controls (Fig. 1d). In the marble-burying test, IP3R2 KO mice buried more marbles than their control littermates (Fig. 1e). These observations demonstrate that IP3R2 KO mice show increased repetitive behaviors.

Although ASD is generally regarded as a neurodevelopmental syndrome, recent studies have shown that dysfunctions in autism risk genes both during early development and in adulthood result in autism-like phenotypes that are reversible in adult animals when the normal functions of these risk genes are restored^33–36^. To determine whether astrocyte-specific IP3R2 knockout in adult mice is sufficient to produce autism-related behaviors, we crossed Aldh1L1-CreER mice, which express Cre recombinase specifically in astrocytes when treated with tamoxifen (Supplementary Fig. 1f, g)^37,38^, with IP3R2^loxp/loxp^ mice, which harbor loxp-flanked IP3R2, to generate Aldh1L1-CreER:IP3R2^loxp/loxp^ mice (hereafter referred to as IP3R2 cKO mice) (Supplementary Fig. 1c, d). The intraperitoneal (i.p.) administration of 75 mg/kg tamoxifen for 7 days in adult IP3R2 cKO mice resulted in a marked reduction in IP3R2 expression (Supplementary Fig. 1e). IP3R2 expression was reduced in astrocytes isolated by fluorescence-activated cell sorting (Supplementary Fig. 1h-k). IP3R2 cKO mice were grossly normal and healthy (exhibiting normal fur color, appetite, and body weight) (data not shown). Furthermore, immunostaining indicated that the morphology and density of astrocytes and neurons showed no difference in the mPFC between IP3R2 cKO and control mice (Supplementary Fig. 5a, b). Biocytin staining further revealed no alterations in the spine density of mPFC layer 5 pyramidal neurons after IP3R2 conditional knockout in astrocytes (Supplementary Fig. 5c, d). These data suggest a specific loss of IP3R2 in astrocytes results in no significant change in astrocyte reactivity and synaptic and neuronal morphology.

To further confirm that astrocyte-specific IP3R2 conditional knockout leads to impairments in astrocyte intracellular Ca^2+^ signals, two-photon Ca^2+^ imaging experiments (Supplementary Fig. 6a, b) were repeated as in IP3R2 KO mice (Supplementary Fig. 2a, b). Similar to IP3R2 KO mice, IP3R2 cKO mice also displayed impairments in astrocyte somata Ca^2+^ signals under both spontaneous (Supplementary Fig. 6c-g) and GPCRs agonist cocktail-induced (Supplementary Fig. 6h-k) conditions. While spontaneous Ca^2+^ signals in astrocytic processes were dramatically reduced in IP3R2 cKO mice (Supplementary Fig. 6c-g), cocktail-induced Ca^2+^ signals were largely unaffected (Supplementary Fig. 6l-n).

Similar to IP3R2 KO mice, IP3R2 cKO mice showed deficits in social interaction in the three-chamber test, as they showed no preference for the mouse-containing cage over the empty cage (Fig. 1f, Supplementary Fig. 3b); IP3R2 cKO mice displayed no alterations in social preference, compared to wild-type mice, as they preferred to socialize with the second stranger mouse to a similar extent (Fig. 1g). No differences were observed in anxiety levels or locomotor activity, as assessed by the EPM test and OFT, between IP3R2 cKO and control mice (Supplementary Fig. 7a-d). In the self-grooming assay and marble-burying test, IP3R2 cKO mice demonstrated repetitive behaviors; compared to wild-type littermates, they spent a longer time self-grooming and buried more marbles (Fig. 1i, j). Unlike IP3R2 KO mice, IP3R2 cKO mice showed no alterations in performance in the NOR test (Fig. 1h and Supplementary Fig. 7e-g). These data indicate that the specific deletion of IP3R2 in adult astrocytes is sufficient to induce two core autism-related phenotypes, namely, social deficits and repetitive behaviors.

The mPFC is critically involved in social behaviors in physiological and pathological conditions^39,40^ and a candidate region for impaired functions in ASD^39,41–43^. To further determine the consequences of selective disturbance of IP3R2 in mPFC astrocytes, an adeno-associated virus (AAV) virus expressing shRNA targeting IP3R2 mRNA under the control of astrocyte-specific GFAP promoter (AAV-GFAP-IP3R2 shRNA) was injected into the mPFC of C57BL/6J mice to specifically knockdown IP3R2 in astrocytes (Supplementary Fig. 8a). Western blotting revealed IP3R2 protein levels were significantly decreased in the mPFC of mice injected with AAV-GFAP-IP3R2 shRNA1 and shRNA2 compared to controls (Supplementary Fig. 8b, c). Behavioral analysis showed that mice injected with IP3R2 shRNAs exhibited deficits in social interaction in the three-chamber test (Fig. 1k, l, Supplementary Fig. 3c), but displayed neither cognitive impairments in the NOR test (Fig. 1m, Supplementary Fig. 9e-g) nor repetitive behaviors in self-grooming assay (Fig. 1n) and marble-burying test (Fig. 1o). No differences were observed in anxiety-like behavior or locomotor activity among all groups (Supplementary Fig. 9a-d). Thus, selective deletion of IP3R2 in mPFC astrocytes recapitulates social deficits observed in IP3R2 KO and cKO mice. Of note, IP3R2 knockdown in mPFC astrocytes produces modest but significant behavioral alterations, as compared to IP3R2 mutant mice, possibly due to the contribution of astrocytic IP3R2 signaling in other brain regions such as the hippocampus to social behaviors^44,45^.

**Fig. 2:**
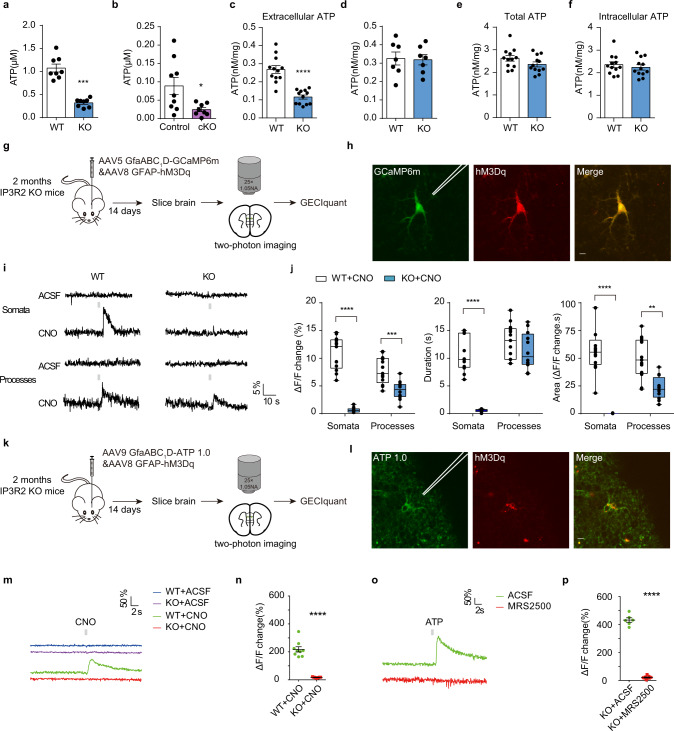
Astrocytic ATP release is impaired in IP3R2 KO mice. **a**, **b** ATP levels in the mPFC of IP3R2 KO (**a** *U* = 0, *P* = 0.0002, *n* = 8) and IP3R2 cKO (**b** *U* = 14, *P* = 0.0360, *n* = 9/8) mice. IP3R2^loxp/loxp^ mice were used as control (**b**). **c**, **d** ATP levels in the culture medium of astrocytes (**c**, *t*_22_ = 6.397, *P* = 0.3 × 10^−5^, *n* = 12) or neurons (**d** *t*_12_ = 0.1578, *P* = 0.8772, *n* = 7) from IP3R2 KO and WT mice. **e**, **f** Total ATP levels (**e** *t*_22_ = 1.620, *P* = 0.1195, *n* = 12) and intracellular ATP levels (**f** *t*_22_ = 0.6959, *P* = 0.4938, *n* = 12) in astrocytes from IP3R2 KO and WT mice. **g** Schematic experimental approach. **h** Two-photon calcium imaging of an hM3Dq&GCaMP6m-expressing astrocyte, with an adjacently placed glass pipette. Scale bar, 5 μm. **i** Representative fluorescence traces from an hM3Dq&GCaMP6m-expressing astrocyte treated with CNO or ACSF in IP3R2 WT and KO mice. A gray line represents the 200 ms of 10 mM CNO application. **j** Summary of the results from all imaged cells (*WT* vs*. KO*, Δ*F*/*F* change, somata*: t*_23_ = 12.473, *P* = 0, processes*: t*_23_ = 4.271, *P* = 0.286 × 10^*−*3^; duration, *somata: t*_23_ = 11.826, *P* = 0, processes: *t*_23_ = 1.305, *P* = 0.2058; area, somata: *t*_23_ = 7.839, *P* = 0, processes: *t*_23_ = 3.239, *P* = 0.3622 × 10^*−2*^; *n* = 13/12 cells from 3 mice each). Box plots present, in ascending order, minimum sample value, first quartile, median, third quartile, and maximum sample value. **k** Schematic experimental approach. **l** Same as (**h**) but for an hM3Dq- and ATP1.0-expressing astrocyte. Scale bar, 5 μm. **m** Same as (**i**) but for an hM3Dq- and ATP1.0-expressing astrocyte. Representative movies are shown as Supplementary Videos 1-4. **n** Summary of the results from all imaged cells (*t*_16_ = 10.79, *P* = 0.5 × 10^−5^, *n* = 9 cells from 3 mice each). **o** Representative fluorescence traces from hM3Dq- and ATP1.0-expressing astrocytes pre-treated with ACSF or 30 μM MRS2500 (a P2Y1R antagonist) in IP3R2 KO mice. **p** Summary of the results from all imaged cells (*t*_13_ = 25.20, *P* = 0.1 × 10^−5^, *n* = 6/9 cells from 3 mice each). Data are presented as mean ± SEM. *P < 0.05, **P < 0.01, ***P < 0.001, ****P < 0.0001. Two-tailed unpaired *t* test (**a**–**f**, **j**, **n**, **p**). Source data are provided as a Source data file.

To characterize the neurobiological mechanisms that underlie autism-related phenotypes in IP3R2 mutant mice, we analyzed the levels of gliotransmitters that are known to be secreted by astrocytes in the mPFC. Interestingly, in vivo microdialysis experiments showed a remarkable reduction in ATP levels in IP3R2 KO mice when compared to control mice (Fig. 2a) but no changes in other gliotransmitters (Supplementary Fig. 10a, b). Similar results were also observed in IP3R2 cKO mice (Fig. 2b and Supplementary Fig. 10c). ATP levels in the hippocampus and striatum were decreased in IP3R2 KO mice as well (Supplementary Fig. 11). Notably, ATP concentrations were markedly lower in the culture medium of astrocytes (Fig. 2c), but not neurons (Fig. 2d), isolated from IP3R2 KO mice, suggesting a specific reduction in astrocytic ATP due to a lack of IP3R2. ATP levels were also assessed in peripheral organs such as the liver, lung, kidney, thymus, and blood that have been reported to show abundant expression of IP3R2 and Aldh1L1^38,46^, and no obvious changes were detected in IP3R2 KO and cKO mice (Supplementary Fig. 12). Together, these results demonstrate that ATP levels are reduced following the deletion of IP3R2 in astrocytes.

To figure out the mechanisms underlying the reduction in extracellular ATP levels, we evaluated the total ATP levels and intracellular ATP levels by lysing the cultured astrocytes from IP3R2 KO mice. We found no change in the total ATP levels (Fig. 2e) or intracellular ATP levels (Fig. 2f), indicating that extracellular ATP levels were selectively reduced (Fig. 2b), which could be due to a decrease in ATP release or an increase in ATP degradation by extracellular ectonucleotidases (ecto-ATPases) or both. To clarify this assumption, we firstly employed chemogenetic approach to express the Gq-coupled receptor hM3Dq under the control of GFAP promoter (AAV8-GFAP::hM3Dq-mCherry) in mPFC astrocytes, allowing their time-restricted activation by clozapine-N-oxide (CNO). The hM3Dq expression was specifically overlapped with the astrocytic marker S100β, suggesting high specificity (Supplementary Fig. 13). To verify that hM3Dq activates astrocytes upon CNO application, we performed two-photon calcium imaging by co-expressing hM3Dq and GCaMP6m in mPFC astrocytes, with a glass pipette placed adjacently to locally apply CNO (10 mM) (Fig. 2g, h). In hM3Dq-expressing astrocytes, CNO application triggered an increased Ca^2+^ signals both in the somata and processes in IP3R2 WT mice (Fig. 2i, j). As expected, CNO failed to trigger Ca^2+^ signals in the somata in IP3R2 KO mice, whereas Ca^2+^ signals in the processes could still be induced (Fig. 2i, j), similar to the effects of a GPCRs agonist cocktail (Supplementary Fig. 2). To directly detect ATP release from astrocytes in real-time, we expressed a newly developed genetically-encoded fluorescent GPCR-activation-based ATP (GRAB_ATP1.0_, hereafter referred to as ATP1.0) sensor which was designed by using human P2Y1R as the scaffold combined with circularly permuted enhanced GFP (data not shown). Under the control of the astrocytic *GfaABC*_*1*_*D* promotor (AAV9-GfaABC_1_D::ATP1.0), the ATP1.0 was specifically expressed in mPFC astrocytes (Supplementary Fig. 14). Taking advantage of the hM3Dq and ATP1.0, we could time-dependently stimulate mPFC astrocytes by local CNO application and monitor released ATP adjacent to cell membrane of the activated astrocyte with two-photon imaging in real-time (Fig. 2k, l). Upon application of CNO, we observed that the ATP release was significantly reduced in IP3R2 KO mice compared to WT mice (Fig. 2m, n). To verify that the ATP1.0 works efficiently in IP3R2 KO mice, we delivered ATP as a positive control. We found that ATP1.0 responded to ATP (2 μM) application, which was completely blocked by the selective P2Y1 receptor antagonist MRS2500 (30 μM) (Fig. 2o, p). Thus, these data demonstrate that the reduced extracellular ATP levels may result from a decrease in ATP release.

An increase in ATP degradation by extracellular ecto-ATPases may also contribute to the reduced extracellular ATP levels. To test this assumption, we screened a variety of ecto-ATPase mRNA levels and found that the mRNA levels of ecto-ATPase ENPP1 and ENTPD3 were significantly increased, paralleled by a comparable increase in protein levels (Supplementary Fig. 15a-d). To determine whether the elevated ecto-ATPase led to the reduced extracellular ATP levels, we applied an ATPase inhibitor ARL67156 (50 μM)^47^ into the medium of cultured astrocytes for 2 days and found that this treatment produced a bigger increase in ATP change in cultured astrocytes from IP3R2 KO mice compared to WT mice (Supplementary Fig. 15e-f), suggesting that an enhanced ecto-ATPase activity may also account for the reduced extracellular ATP levels. Together, these data indicate that both decreased ATP release and increased ATP hydrolysis may work together to reduce the extracellular ATP levels in IP3R2 mutant mice.

**Fig. 3:**
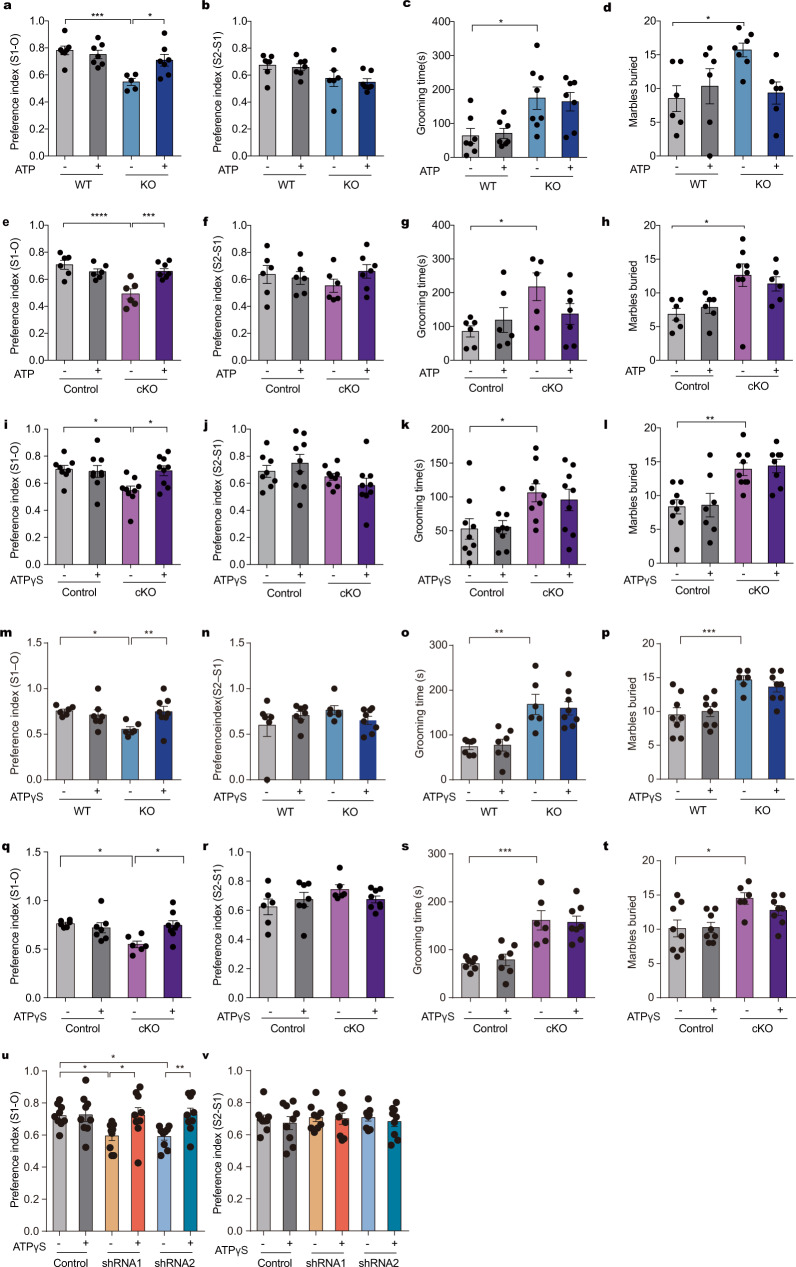
Rescue of social behaviors by acute ATP or ATPγS treatment in IP3R2 mutant mice. **a**, **b** Intraperitoneal (i.p.) injection of 125 mg/kg ATP restored social interaction in IP3R2 KO mice (**a** *F*_3,23_ = 8.876, *P* = 0.0004; KO_vehicle_ vs. KO_ATP_, *P* = 0.0155; **b** *F*_3,23_ = 2.925, *P* = 0.0553; *n* = 7,7,6,7). **c**, **d** i.p. ATP treatment did not affect repetitive behaviors (**c** *F*_3,25_ = 5.323, *P* = 0.0056, *n* = 7,7,8,7; **d** *F*_3,21_ = 3.372, *P* = 0.0377, *n* = 6,6,7,6). **e**, **f** Same as (**a**, **b**) but for IP3R2 cKO mice (**e** *F*_3,21_ = 13.95, *P* = 0.32 × 10^−4^; cKO_vehicle_ vs. cKO_ATP_, *P* = 0.371 × 10^−3^; **f** *F*_3,21_ = 0.7249, *P* = 0.5484; *n* = 6,6,6,7). **g**, **h** Same as (**c**, **d**) but for IP3R2 cKO mice (**g** *F*_3,20_ = 2.761, *P* = 0.0689, *n* = 6,6,5,7; **h** *F*_3,22_ = 4.761, *P* = 0.0105, *n* = 6,6,8,6). **i**, **j** Intracerebroventricular (i.c.v.) injection of ATPγS (50 µM) restored social interaction in IP3R2 cKO mice (**i** *F*_3,31_ = 4.238, *P* = 0.0127; cKO_vehicle_ vs. cKO_ATPγs_, *P* = 0.0339; **j** *F*_3,31_ = 2.059, *P* = 0.126; *n* = 8,9,9,9). **k**, **l** i.c.v. ATPγS treatment did not affect repetitive behaviors (**k** *F*_3,32_ = 3.942, *P* = 0.0168, *n* = 9; **l** *F*_3,29_ = 8.058, *P* = 0.0005, *n* = 9,7,9,8). **m**, **n** Same as (**j**) but for intra-mPFC injection in IP3R2 KO mice (**m** *F*_3,23_ = 3.401, *P* = 0.0348; KO_vehicle_ vs. KO_ATPγS_, *P* = 0.0099; **n** *F*_3,23_ = 0.9799, *P* = 0.4194; *n* = 6,7,6,8). **o**, **p** Intra-mPFC ATPγS treatment did not affect repetitive behaviors (**o** *F*_3,24_ = 11.87, *P* = 0.58 × 10^−4^, *n* = 7,7,6,8; **p** *F*_3,26_ = 9.106, *P* = 0.0003, *n* = 8,8,6,8). **q**, **r** Same as (**m**, **n**) but for IP3R2 cKO mice (**q** *F*_3,23_ = 4.721, *P* = 0.0104; cKO_vehicle_ vs. cKO_ATPγs_, *P* = 0.0186; **r** *F*_3,23_ = 1.314, *P* = 0.2939; *n* = 6,7,6,8). **s**, **t** Same as (**o**, **p**) but for IP3R2 cKO mice (**s** *F*_3,24_ = 13.62, *P* = 0.22 × 10^−4^, *n* = 7,7,6,8; **t** *F*_3,26_ = 4.789, *P* = 0.0087, *n* = 8,8,6,8). **u**, **v** Same as (**m**, **n**) but for IP3R2 knockdown mice (**u** *F*_5,48_ = 3.954, *P* = 0.0044; shRNA1_vehicle_ vs. shRNA1_ATPγs_, *P* = 0.0108; shRNA2_vehicle_ vs. shRNA2_ATPγs_, *P* = 0.0063; **v** *F*_5,48_ = 0.2194, *P* = 0.9525; *n* = 9). Each data point represents an individual mouse. Data are presented as mean ± SEM. *P < 0.05, **P < 0.01, ***P < 0.001, ****P < 0.0001. One-way ANOVA with Tukey’s (**a**–**l**, **n**–**t**, **v**) or Fisher’s LSD (**m**, **u**) multiple comparison post hoc test. Source data are provided as a Source data file.

To test whether ATP reduction is responsible for the autism-like phenotypes in IP3R2 mutant mice, a single i.p. injection of ATP at a dose of 62.5 or 125 mg/kg was applied 30 min prior to behavioral tests. The concentrations of ATP were chosen based on previous reports^21^, which could increase ATP abundance in the brain (Supplementary Fig. 16). Consistent with the aforementioned results (Fig. 1a, b), IP3R2 KO mice displayed impaired social interaction (Fig. 3a) but normal social preference (Fig. 3b) in the three-chamber social interaction test (Supplementary Fig. 3e). Notably, ATP treatment dramatically increased social interaction in a dose-dependent manner in IP3R2 KO mice (Supplementary Figs. 3d, 17a) to an extent similar to that in wild-type littermates (Fig. 2a), with the social preference largely unaltered (Supplementary Fig. 17b). IP3R2 KO mice demonstrated increased repetitive behaviors compared to wild-type controls, as revealed by the self-grooming test (Fig. 3c) and marble-burying test (Fig. 3d); however, these behavioral abnormalities were not reversed by ATP treatment (Fig. 3c, d). ATP administration had no effect on either anxiety-like behavior, as assessed by the EPM test, or locomotion, as assessed by the OFT, in IP3R2 KO mice (Supplementary Fig. 18a-c). These results demonstrate that ATP treatment partially rescues autism-like behaviors, i.e., social deficits, in IP3R2 KO mice.

We then examined whether the administration of ATP can ameliorate autism-like behaviors in IP3R2 cKO mice. Acute treatment with ATP (125 mg/kg, i.p.) completely restored social interaction in IP3R2 cKO mice without affecting social preference (Fig. 3e, f, Supplementary Fig. 3f). Moreover, the intracerebroventricular (i.c.v.) infusion of ATPγS (25 µM), a nonhydrolyzable ATP analog^21^, dramatically rescued social deficits in IP3R2 cKO mice (Fig. 3i, j, Supplementary Fig. 3g), ruling out the possible contribution of ATP hydrolysis products and ATP effects on peripheral tissues. The effects of ATP and ATPγS on repetitive behavior, as assessed by the self-grooming test and marble-burying test, were negligible between wild-type and IP3R2 cKO mice (Fig. 3g, h, k, l). Treatment with either ATP or ATPγS had no sedative or anxiolytic effects (Supplementary Fig. 18d-i). These results show that a single ATP or ATPγS treatment can also restore impaired social interaction in IP3R2 cKO mice. Taken together, our findings suggest a causal link between astrocytic ATP deficiencies and social impairments in IP3R2 mutant mice.

To determine whether astrocytic ATP decrease in the mPFC contributes to social impairments in IP3R2 mutant mice, intra-mPFC infusion of ATPγS (50 µM, 1 µL) was applied 30 min before the behavioral tests. As expected, intra-mPFC infusion of ATPγS rescued social impairments but not repetitive behaviors in IP3R2 KO (Fig. 3m–p, Supplementary Fig. 3h) and IP3R2 cKO (Fig. 3q–t, Supplementary Fig. 3i) mice, without producing sedative or anxiolytic effects (Supplementary Fig. 18j-o). Furthermore, the social deficits of mice treated with AAV-GFAP-IP3R2 shRNA1 and shRNA2 could be restored by ATPγS (Fig. 3u, v, Supplementary Fig. 3j), without sedative or anxiolytic effects (Supplementary Fig. 18p-r). These data implicate the mPFC as a potential locus for social impairments in IP3R2 mutant mice.

**Fig. 4:**
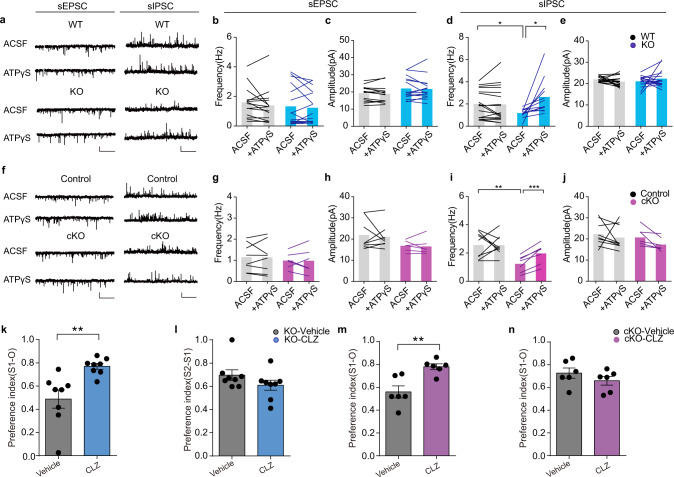
IP3R2 mutant mice exhibit impaired GABAergic neurotransmission, which can be restored by ATPγS treatment. **a** sEPSC and sIPSC recordings with and without ATPγS (25 µM) treatment in mPFC layer 5 pyramidal neurons from WT and IP3R2 KO slices. Scale bars: 20 pA, 2 s. **b**–**e** Bar graphs showing sEPSC and sIPSC frequency (**b**, **d**: *WT*_ACSF_ vs*. KO*_ACSF_, two-tailed unpaired *t*-test, *t*_27_ = 2.173, *P* = 0.0387, *n* = 17/12; *WT*_ACSF_ vs*. WT*_ATPγS_, two-tailed paired *t*-test, *t*_16_ = 0.3945, *P* = 0.6984, *n* = 17; *KO*_ACSF_ vs*. KO*_ATPγS_, two-tailed paired *t*-test, *t*_11_ = 2.905, *P* = 0.0143, *n* = 12) and amplitude (**c**, **e**) in WT and IP3R2 KO slices with and without ATPγS treatment. IP3R2 KO mice exhibited impaired GABAergic neurotransmission that was restored by ATPγS treatment. **f** Same as (**a**) but for IP3R2 cKO slices. Scale bars: 20 pA, 2 s. **g**–**j** Bar graphs showing sEPSC and sIPSC frequency (**g**, **i** *control*_ACSF_ vs*. cKO*_ACSF_, two-tailed unpaired *t*-test, *t*_14_ = 3.163, *P* = 0.0069, *n* = 7/9; *control*_ACSF_ vs. *control*_*ATPγs*_, two-tailed paired *t*-test, *t*_8_ = 0.1744, *P* = 0. 8659, *n* = 9; *cKO*_ACSF_ vs. *cKO*_*ATPγs*_, two-tailed paired *t*-test, *t*_6_ = 6.462, *P* = 0.0007, *n* = 7) and amplitude (**h**, **j**) in control and IP3R2 cKO slices. **k**, **l** Rescue of social behaviors in IP3R2 KO mice upon acute clonazepam (CLZ, 0.0625 mg/kg, i.p.) treatment in the three-chamber test (**k** *U* = 3, *P* = 0.0011; **l** *t*_14_ = 1.399, *P* = 0.1836, *n* = 8). **m**, **n** Same as (**k**, **l**) but for IP3R2 cKO mice (**m** *t*_10_ = 3.757, *P* = 0.0037; **n** *t*_10_ = 1.153, *P* = 0.2758, *n* = 6). Each data point represents an individual mouse (**k**–**n**). Data are presented as the mean ± SEM. Two-tailed unpaired *t* test (**l**–**n**). Mann–Whitney U-test (**k**). *P < 0.05, **P < 0.01, ***P < 0.001. Source data are provided as a Source data file.

The observations that the behavioral and physiological deficits in animal models of ASD are reversible upon pharmacological or genetic manipulation, together with the synaptic theory of autism, strongly suggest an ongoing synaptopathy as the underlying cause of ASD^35,48–50^. To test this hypothesis in our IP3R2 mutant mouse model of autism, we compared the synaptic properties of pyramidal neurons in the mPFC of wild-type and IP3R2 mutant mice. Whole-cell recordings from layer 5 pyramidal neurons showed that the amplitude and frequency of spontaneous excitatory postsynaptic currents (sEPSCs) were comparable between wild-type and IP3R2 KO mice (Fig. 4a, b, c), indicating that the glutamatergic neurotransmission of pyramidal neurons was not affected by IP3R2 deletion, which is consistent with previous studies of the hippocampus^25,31^. However, the frequency (Fig. 4a, d), but not the amplitude (Fig. 4a, e), of spontaneous inhibitory postsynaptic currents (sIPSCs) was dramatically decreased in IP3R2 KO mice compared to wild-type littermates, suggesting reduced GABAergic inputs to pyramidal neurons. Similar results were also observed in IP3R2 cKO mice, as we detected a marked decrease in the frequency (Fig. 4f, i), but not the amplitude (Fig. 4f, j), of sIPSCs. Again, both the frequency and amplitude of sEPSCs were unaltered by the astrocyte-specific deletion of IP3R2 (Fig. 4f–h). Notably, deficits in social interaction were ameliorated by the acute application of the GABA_A_R agonist clonazepam (0.0625 mg/kg, i.p.) both in IP3R2 KO mice (Fig. 4k, l, Supplementary Fig. 3k) and cKO mice (Fig. 4m, n, Supplementary Fig. 3l), suggesting that GABAergic dysfunction may underlie autism-like phenotypes in IP3R2 mutant mice. To examine whether impaired GABAergic transmission in IP3R2 mutant mice is caused by a reduction in astrocytic ATP, we tested the rescuing effect of ATPγS at a concentration (25 µM) that does not affect synaptic transmission in wild-type mice (Fig. 4a–j). The perfusion of ATPγS completely reversed the decrease in sIPSC frequency in IP3R2 KO (Fig. 4a, d) and cKO (Fig. 4f, i) slices. Together, these findings demonstrate that the deletion of IP3R2 in astrocytes leads to synaptic deficits that may underlie the pathogenesis of autism-like behaviors, both of which are normalized by ATP treatment.

**Fig. 5:**
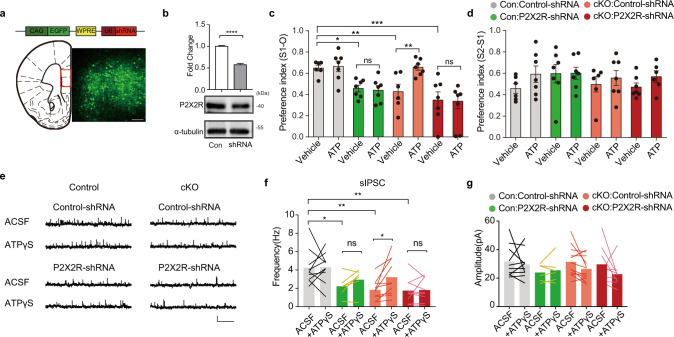
P2X2 receptors in the prefrontal cortex mediate the beneficial effects of ATP on ASD-like behaviors. **a** A schematic of the AAV vector carrying P2X2R-shRNA (top). The viral expression of P2X2R-shRNA (bottom right) and the injection site in the mPFC (bottom left, red box, scale bar: 100 µm). **b** Western blot showing P2X2R knockdown in the mPFC with AAV-P2X2R-shRNA (two-tailed unpaired *t*-test, *t*_6_ = 12.71, *P* = 0.15 × 10^−4^, *n* = 4). **c**, **d** i.p. injection of ATP (125 mg/kg) rescued social interaction in IP3R2 cKO mice, and this effect was blocked by P2X2R knockdown (**c** *F*_7,45_ = 6.973, *P* = 0.7 × 10^−5^; *Con:Control-shRNA*_*vehicle*_ vs. *Con:P2X2R-shRNA*_*vehicle*,_ *P* = 0.0142; *Con:Control-shRNA*_*vehicle*_ vs. *cKO:Control-shRNA*_*vehicle*_, *P* = 0.0066; *Con:Control-shRNA*_*vehicle*_ vs. *cKO:P2X2R-shRNA*_*vehicle*_, *P* = 0.0002; *Con:P2X2R-shRNA*_*vehicle*_ vs. *Con:P2X2R-shRNA*_*ATP*_, *P* = 0.8171; *cKO:Control-shRNA*_*vehicle*_ vs*. cKO:Control-shRNA*_*ATP*_, *P* = 0.0037; *cKO:P2X2R-shRNA*_*vehicle*_ vs*. cKO:P2X2R-shRNA*_*ATP*_, *P* = 0.8931; **d** *F*_7,45_ = 0.8109, *P* = 0.5830; *n* = 6,7,7,7,6,7,7,7). **e** sIPSC recordings with and without ATPγS (25 µM) treatment in mPFC layer 5 pyramidal neurons from control and IP3R2 cKO mice injected with control-shRNA or P2X2R-shRNA. **f**, **g** Bar graphs showing sIPSC frequency (**f** *Con:Control-shRNA*_*ACSF*_ vs. *Con:P2X2R-shRNA*_*ACSF*_, two-tailed unpaired *t*-test, *t*_14_ = 2.381, *P* = 0.0320, *n* = 10/6; *Con:Control-shRNA*_*ACSF*_ vs. *cKO:control-shRNA*_*ACSF*_, two-tailed unpaired *t*-test, *t*_18_ = 3.539, *P* = 0.0023, *n* = 10; *Con:Control-shRNA*_*ACSF*_ vs. *cKO:P2X2R-shRNA*_*ACSF*_, two-tailed unpaired *t*-test, *t*_17_ = 3.273, *P* = 0.0045, *n* = 9/10; *Con:P2X2R-shRNA*_*ACSF*_ vs. *Con:P2X2R-shRNA*_*ATPγs*_, two-tailed paired *t*-test, *t*_5_ = 2.001, *P* = 0.1018, *n* = 6; *cKO:Control-shRNA*_*ACSF*_ vs. *cKO:Control-shRNA*_*ATPγs*_*, t*wo-tailed paired *t*-test, *t*_9_ = 2.777, *P* = 0.0215, *n* = 10; *cKO:P2X2R-shRNA*_*ACSF*_ vs. *cKO:P2X2R-shRNA*_*ATPγs*_, two-tailed paired *t*-test, *t*_8_ = 0.1624, *P* = 0.8750, *n* = 9) and amplitude (**g**) with or without ATPγS (25 µM) treatment in mPFC layer 5 pyramidal neurons from control and IP3R2 cKO mice injected with control-shRNA or P2X2R-shRNA. P2X2R knockdown prevented the enhancing effect of ATPγS (25 µM) on sIPSC frequency in IP3R2 cKO slices. Scale bars: 20 pA, 2 s. Each data point represents an individual mouse (**c**, **d**). Data are presented as the mean ± SEM. *P < 0.05, **P < 0.01, ***P < 0.001, ****P < 0.0001. ns, no significant difference. One-way ANOVA with Fisher’s LSD multiple comparison post hoc test (**c**, **d**). Source data are provided as a Source data file.

Previous studies have reported that ATP regulates GABAergic synaptic transmission and behaviors through P2X2 receptors (P2X2Rs)^21,51^. Based on these findings, we tested the assumption that P2X2R mediates the anti-autistic effects of ATP in IP3R2 mutant mice. To this end, an AAV expressing P2X2R-shRNA was bilaterally injected into the mPFC of IP3R2 cKO and control mice to silence P2X2R (Fig. 5a, b) without potential non-specific off-target effects (Supplementary Fig. 19). We found that knockdown of P2X2R not only induced social deficits in WT mice, but also prevented the anti-autistic effects of ATP in IP3R2 cKO mice in the three-chamber test (Fig. 5c, d, Supplementary Fig. 3m). Furthermore, P2X2R knockdown impaired GABAergic neurotransmission in WT mice and prevented the ATPγS-induced increase in sIPSC frequency in IP3R2 cKO slices (Fig. 5e–g). Together, these results demonstrate that P2X2R in the mPFC is required for the therapeutic effects of ATP, possibly by enhancing GABAergic synaptic transmission.

## Discussion

Here, we show that astrocyte calcium signaling dysfunction can result in ASD-like behaviors in mice. The alterations of astrocytes in the brains of autism patients and animal models have been noted repeatedly^6–13,16,17^. However, the roles of astrocytes in ASD remains unclear. Here we also report that mice with astrocyte-specific IP3R2 deficiency show ASD-like behaviors, including social interaction deficits and repetitive behavior, highlighting the usefulness of IP3R2 mutant mice as a model to study astrocyte dysfunctions in ASD models.

Astrocytes can release a variety of synaptic transmitters and modulators, including but not limited to glutamate, D-serine, ATP/adenosine, GABA and lactate through calcium-dependent and -independent signaling pathways^52–55^. We found that not all gliotransmitters were affected equally in IP3R2 mutant mice; instead, only ATP deficiency was observed in the absence of IP3R2, consistent with past work^56^. The reasons underlying this observation are currently unclear, which could be multiple. For example, the synthesis, release, reuptake, and degradation of different gliotransmitters may be differentially regulated in the absence of IP3R2, leading to an overall change below detection limit. In this study, we found the combination of decreased ATP release and increased ATP degradation may account for the lower extracellular ATP levels in IP3R2 mutant mice, suggesting that IP3R2-mediated signaling regulates ATP levels through distinct pathways. This may hold true for IP3R2-mediated regulation of other gliotransmitters as well. Since ATP acts both as an energy currency as well as a neurotransmitter, we cannot exclude the possibility that the seemingly unchanged intracellular ATP levels may result from a concurrent increase (or decrease) in ATP synthesis and consumption. Future studies on the mechanisms underlying the regulation of ATP homeostasis, particularly that of decreased ATP release and increased ATPase expression in IP3R2 mutant mice, may help clarify this assumption^57^. Another possibility is the ambient levels of gliotransmitters such as glutamate, GABA, and D-serine may be regulated in an IP3R2-independent manner^58–60^. This seems very likely because astrocyte calcium signals, particularly those in processes, were not completely abolished in IP3R2 mutant mice^27^ and because the release of gliotransmitters primarily occurs in astrocytic processes^61^.

We identified P2X2 receptors as the mediator of astrocyte-derived ATP in regulating dysfunction of GABAergic transmission and ASD-like behaviors in IP3R2 mutant mice. Astrocyte-derived ATP has been reported to modulate synaptic and behavior functions by binding to various P2X and P2Y receptors directly, or by binding to A1 receptors after being converted to adenosine by extracellular ecto-ATPases^21,51,56,62–65^. While we cannot rule out the possibility that other purine receptors are involved, the observation that knockdown of P2X2 receptors prevents the synaptic and behavior effects of ATP strongly suggests that P2X2 may play a critical role in these processes. P2X receptor subunits are widely expressed in the brain both in neurons and glia, and P2X2 receptors are most abundant in neurons^66^. Of note, the expression of P2X2 has been reported to be preferentially located at the periphery of the postsynaptic specialization^67^, indicating that P2X2 receptors are suitable for sensing ATP released from astrocytic processes. Furthermore, presynaptic P2X2 receptors facilitate glutamate release onto GABAergic interneurons^68,69^, perturbations of which may decrease interneuron activity, which may in turn, lead to a decrease in GABAergic neurotransmission. Consistent with this notion, our study suggests GABAergic transmission as the potential synaptic pathophysiology that underlies ASD-like behaviors in IP3R2 mutant mice, as both the synaptic and behavioral deficits of IP3R2 mutant mice were partially or completely normalized by ATP application and by enhancing GABAergic transmission. These findings are in line with the synaptopathy hypothesis of ASD^35,48–50,70^. In multiple animal models of ASD, such as the Shank mutant mice^49^, the behavioral and synaptic deficits can also be partially or completely reversed through genetic restoration or pharmacological manipulation in adulthood, suggesting that ASD may be ameliorated by appropriate treatments^48^. Of note, ATP application rescued impaired social interaction and synaptic deficits but not repetitive behaviors in mice, suggesting that ATP-independent mechanisms may also play a role^71^, which awaits further studies. Interestingly, some studies have reported a high comorbidity of ASD and depression^72–74^, suggesting that there may be some pathophysiological mechanisms shared by these two diseases. Together with our previous findings that IP3R2 KO mice also demonstrate depressive-like behaviors due to a deficiency of ATP^21^, we hypothesise that astrocyte-derived ATP is involved in the pathophysiology of both disorders in mouse models.

In summary, our findings suggest a role for astrocytes in the pathogenesis of ASD-like behavior and identify astrocyte-derived ATP as a potential molecular player in mice with IP3R2 deletion.

## Methods

### Animals and drug treatment

Mice were housed in standard laboratory cages (4–5 per cage) at temperature 24 ± 1°C, humidity ~60%, maintained on a 12-h light/dark cycle with lights on at 8:00 a.m., and provided food and water ad libitum. Adult C57BL/6J mice (aged 8–10 weeks) were from the Laboratory Animal Center, Southern Medical University.

Both IP3R2 null mutant mice (IP3R2 KO) and IP3R2 floxed mice were gifts from Dr. Ju Chen. IP3R2 KO mice (IP3R2 KO) were generated by crossing germline heterozygous-null knockout (IP3R2^+/−^) mice. The offspring were genotyped by tail DNA PCR. The PCR primers for the wild-type allele were 5′-GCTGTGCCCAAAATCCTAGCACTG-3′ and 3′-CATGCAGAGGTCGTGTCAGTCATT-5′. The mutant allele specific primers were 5′-AGTGATACAGGGCAAGTTCATAC-3′ and 3′-AATGGGCTGACCGCTTCCTCGT-5′. The genotyping primers for IP3R2 floxed mice PCR were 5′-GCTGTGCCCAAAATCCTAGCACTG-3′ and 3′-CATGCAGAGGTCGTGTCAGTCATT-5′.

Aldh1l1-CreER mice were generated by the GemPharmatech co. Ltd (Nanjing, China). Briefly, Aldh1l1-CreER knock-in mice were generated via the CRISPR/Cas9 system. Cas9 mRNA, sgRNA and donor plasmid were coinjected into zygotes. sgRNA (CCAGGTCTTGTCCCCAATACTGG)-directed Cas9 endonuclease cleavage near the stop codon created a DSB (double-strand break). Such breaks were repaired and resulted in 2A-CreERT2 insertion before the stop codon of the *Aldh1l1* gene. The mice were screened by PCR analysis and sequencing validation.

To evaluate the expression patterns of Cre recombinase in Aldh1l1-CreER mice, we crossed the Aldh1l1-CreER mice with a fluorescent reporter line (Ai14, Jackson Laboratory, Stock No: 007914).

To induce astrocyte-specific IP3R2 deletion in a temporally controlled manner, we crossed IP3R2^flox/flox^ mice with Aldh1l1-CreER mice. Then, the double heterozygous mice were crossed to generate Aldh1l1::CreER;IP3R2^flox/flox^ mice (mice with astrocyte-specific IP3R2 depletion) and control mice.

All experiments were conducted in accordance with the Regulations for the Administration of Affairs Concerning Experimental Animals (China), that were approved by Animal Care and Use Committee of Southern Medical University and Animal Facility at the Laboratory Animal Center, Southern Medical University, China. At 8 weeks of age, the animals received injections of either saline (i.p.), ATP (disodium salt, 125 mg/kg body weight, i.p., Sigma-Aldrich, #A7669), ATPγS (tetralithium salt, 50 µM, i.c.v., Sigma-Aldrich, #A1388), or clonazepam (CLZ, 0.0625 mg/kg body weight, i.p., Sigma-Aldrich, #C-907).

### Tamoxifen treatment

Tamoxifen was used to induce the expression of the Cre-ER fusion protein. Tamoxifen (Sigma-Aldrich, #T5648) was dissolved in 10% ethanol/90% sunflower oil (Sigma-Aldrich, #S5007) (v/v) at a final concentration of 10 mg/ml. Adult mice (P60) were intraperitoneally (i.p.) treated with tamoxifen (3 mg/40 g body weight), once a day for 7 consecutive days. Experiments were performed 2 weeks after the last dose of tamoxifen.

### Behavioral test

The mice used for all behavioral tests were 8–10 weeks except specifically described. Only male animals were used for behavioral experiments.

### Self-grooming test

Wild-type and IP3R2 knockout mice were individually placed in a new Plexiglas cage (30 × 30 × 35 cm). Each mouse was habituated to the empty cage for 10 min, and then the cumulative time spent grooming all body regions over 10 min was determined.

### Marble-burying task

Mice were placed individually in a Plexiglas cage with 5 cm of fresh bedding. Twenty black marbles were prearranged in the cage in 4 evenly spaced rows of 5 marbles each. The task was conducted for 30 min. Then, the number of buried marbles was counted. A marble was considered buried if more than half of it was covered with bedding.

### Three-chamber test

A three-chamber arena was used to assess social approach and social novelty preference. After a 10-min habituation period in the middle chamber, each mouse was allowed to explore all of the empty chambers for another 10 min. Then, an unfamiliar mouse (stranger 1) enclosed in a wire cage was placed in one of the two side chambers while an identical empty wire cage was placed in another side chamber. Immediately after the mouse was placed in the center chamber, the side doors were opened and the mouse was allowed to explore for 10 min. Then, a new unfamiliar mouse (stranger 2) was placed in the empty wire cage, and the test mouse was examined for an additional 10 min. In this period, social novelty preference was assessed. The time spent in each side chamber was recorded in an automated manner. The time spent sniffing stranger 1, stranger 2, and the empty wire cage was manually scored.

### Elevated plus-maze test

The elevated plus-maze test consisted of four arms (30 × 5 cm) with two open arms without walls and two closed arms with 15 × 25-cm high walls. Each mouse was placed in the center of the elevated plus-maze facing an open arm. Over 5 min, the time spent in each arm was recorded by using EthoVision 11.0 software. The maze was cleaned between sessions using 20% ethanol.

### Open field test

The open field apparatus was a rectangular chamber (40 × 40 × 30 cm) made of gray polyvinyl chloride. Each mouse was gently placed in the center of the testing chamber and recorded for a 5-min period of free movement, which was monitored by an automated video tracking system. A digitized image of the path was recorded and analyzed automatically using EthoVision 11.0 software.

### Novel object recognition (NOR) test

The NOR test was performed as previously described^75^. The NOR apparatus consisted of a rectangular chamber (33 × 33 × 20 cm) that was made of black polyvinyl chloride. Each mouse was habituated to the empty arena for 5 min one day before the familiarization session. During the familiarization session, two identical objects (a tower of Lego bricks and a Falcon tissue culture flask filled with sand) were placed 5 cm away from the walls, and the mice were allowed to explore each object freely until 20 s of total exploration time was reached or until the 10-min period was over. Twenty-four hours later, one familiar object and one novel object were placed sequentially in the arena, and the experiment was terminated when 20 s of exploration of both objects was reached or when the 10-min period was over. The discrimination ratio was calculated to measure the recognition memory of each mouse. The discrimination ratio was computed as follows: the time spent exploring the novel object divided by the total exploration time.

### Stereotaxic microinjection and cannula implantation

The mice were anesthetized and fixed in a stereotaxic apparatus. A hole was drilled at specific *x* and *y* coordinates based on the position of bregma. A Hamilton syringe fitted with a 33-gauge needle was lowered into the mPFC (AP = +1.78, ML = 0.35, DV = 2.55), and 0.3 µl of AAV virus was delivered at 0.1 µl/min. The injection needle was withdrawn 10 min after the infusion. Behavioral tests were performed 2 weeks after AAV injections.

The AAV8-GFAP-hM3Dq-mCherry and ATP1.0 (AAV9-GfaABC1D-ATP1.0) were purchased from Vigenebio Biosciences Co. (Jinan, China). The AAV5-GfaABC1D-GCaMP6m, GFAP-IP3R2 shRNAs and P2X2-shRNA was generated by Shanghai SunBio Biomedical Technology Co. (Shanghai, China).

The sequence of Control-shRNA was 5′-TTCTCCGAACGTGTCACGT-3′. The sequence of GFAP-IP3R2 shRNA1 was 5′-GTCCCAGATCGGCTATGATAT-3′. The sequence of GFAP-IP3R2 shRNA2 was 5′-GCATCTCAATCTGTTCCTAAC-3′. The sequence of GFAP-IP3R2 shRNA3 was 5′-GCAAGTACAGAATGGACTTGG-3′. These shRNAs were ligated into an AAV2/8 vector expressing EGFP. The sequence of P2X2-shRNA was 5′-GCAGGGAAATTCAGTCTCATT-3′. The sequence of the control-shRNA was TTCTCCGAACGTGTCACGT. These shRNAs were ligated into an AAV2/8 vector expressing EGFP.

For intracerebroventricular and intra-mPFC infusion, a guide cannula was unilaterally implanted into the lateral ventricles (AP = −0.2 mm; ML = 1.2 mm; DV = 2.0 mm) or mPFC (AP = 1.8 mm; ML = 0.3 mm; DV = 2.6 mm). After 1-week recovery, the behavioral tests were performed 30 min after infusion. ATPγS (tetralithium salt, 50 µM, Sigma-Aldrich, # A1388) were delivered using an automatic injector (Stoelting, #53311).

### Western blot analysis

Brain tissues were lysed in ice-cold lysis buffer (Cat. No. 11836170001, Roche) containing 1 mmol/l protease inhibitor (PMSF). The samples were then centrifuged for 30 min at 16,000 × *g* at 4 °C, and the supernatant was collected and quantified with the Microplate BCA Protein Assay Kit (#23227, Thermo). The protein samples were separated by SDS-PAGE (10% polyacrylamide gels for IP3R2 and 12% polyacrylamide gels for P2x2) and transferred to PVDF membranes (Millipore). The membranes were blocked with 5% defatted milk at room temperature for 1 h and then incubated overnight with a primary antibody (polyclonal rabbit anti-IP3R2, 1:500, a gift from Professor Ju Chen; polyclonal rabbit anti-P2X2, 1:1000, Abcam, ab48864; polyclonal rabbit anti-ENTPD3 antibody, 1:1000, Proteintech, 13021-1-AP; polyclonal rabbit anti-ENPP1 antibody, 1:1000, Cell Signaling Technology, 2061) at 4 °C. Antibody binding was detected by incubation with an HRP-conjugated secondary antibody (1:10,000, ZB-2305, ZSGB-Bio) at room temperature for 1 h. The protein expression levels were evaluated by quantifying the gray density of the western blot bands with AlphaEaseRFC software (Alpha Innotech Corporation). All samples were normalized to internal controls.

### Microdialysis

Mice (8–10 weeks) were anesthetized and prepared for stereotaxic surgery (Stoelting). A guide cannula (CMA/7, CMA) was implanted into the mPFC. The dummy was removed before inserting the microdialysis probe. The microdialysis probe (CMA/7, CMA) was inserted through the guide cannula and mounted to a CMA microinjection pump (CMA 402). ASCF was continuously perfused through the microdialysis probe at a constant flow rate of 1 µl/min. Samples were automatically collected by using a microfraction collector (CMA 142).

### HPLC-ECD analysis

HPLC analysis was performed to determine the levels of aspartate, glutamate, serine, glutamine, glycine, and GABA. The HPLC system was obtained from Sykam. The mobile phase of HPLC was 90:10 grade water:acetonitrile (consisting of 0.74 mmol/L sodium 1-octanesulfonate, 80 mmol/L monosodium phosphate, 0.027 mmol/L EDTA, and 2 mmol/L KCL), with the pH adjusted to 3.0 by phosphoric acid. An Antec Decade II SD electrochemical detector (VT-03 vitreous carbon working electrode) was used with a Water Xselect HSS T3 chromatographic column (50 mm × 2.1 mm, 2.5 µm) and 0.20 mL/min isocratic elution at 35 °C. The column was connected to an ADF filter (0.05 Hz) and a pulse damper (working voltage: +0.56 V, testing time: 15 min). A 20-µl sample was injected for analysis.

### ATP assay

A bioluminescent ATP assay kit (Promega, TB627) was used to determine the ATP levels^21^. In brief, the ectonucleotidase inhibitor 6-N, N-diethyl-β-γ-dibromomethylene-adenosine-5-triphosphate FPL 67156 (ARL 67156 trisodium salt, 100 µM, Sigma-Aldrich, #A265) was added to the sample to decrease ATP hydrolysis, and a 50-µl sample was added to 50 µl of ATP assay mix containing luciferase-luciferin buffer. Luminescence was measured 12 min later with a luminometer (PerkinElmer, Victor X3). A calibration curve was obtained from serial dilutions of an ATP standard.

### Slice preparation

Male mice (aged 8 weeks) were anesthetized with pentobarbital and decapitated, and the brains were quickly removed and placed in ice-cold oxygenated modified ACSF containing 195 mM sucrose, 2 mM KCl, 0.2 mM CaCl_2_, 12 mM MgSO_4_, 1.3 mM NaH_2_PO_4_, 26 mM NaHCO_3_, and 10 mM glucose. Coronal mPFC slices (300 µm) were prepared using a VT-1200S vibratome (Leica, Germany), subsequently transferred to a storage chamber containing normal ACSF (126 mM NaCl, 3.0 mM KCl, 1.25 mM NaH_2_PO_4_, 2.0 mM CaCl_2_, 1.0 mM MgSO_4_, 26 mM NaHCO_3_, and 10 mM glucose), incubated for 30 min at 34 °C for recovery and subsequently incubated at room temperature (25 ± 1 °C) for an additional 2–8 h. All solutions were saturated with 95% O_2_/5% CO_2_ (vol/vol).

### Electrophysiological recordings

The slices were placed in a recording chamber that was perfused (2 ml/min) with ACSF at 32–34 °C. Whole-cell patch-clamp recordings were obtained under IR-DIC visualization (Zeiss, Axioskop 2). The pipettes were pulled with a micropipette puller (P-97, Sutter instrument) with a resistance of 3–6 MΩ. After establishing the whole-cell configuration, layer 5 pyramidal neurons were held at −70 and 0 mV to record sEPSCs and sIPSCs, respectively. To record sEPSCs, glass pipettes were filled with a solution containing 130 mM potassium gluconate, 20 mM KCl, 10 mM HEPES buffer, 4 mM Mg-ATP, 0.3 mM Na-GTP, 10 mM disodium phosphocreatine and 0.2 mM EGTA, pH adjusted to 7.2 with KOH, 290 mOsm. For sEPSC recording, the GABA_A_ receptors were blocked with 20 µM bicucullinemethiodide (BMI). For sIPSC recording, pipettes were filled with an intracellular solution containing 110 mM Cs_2_SO_4_, 0.5 mM CaCl_2_, 2 mM MgCl_2_, 5 mM EGTA, 5 mM HEPES, 5 mM TEA, 5 mM Mg-ATP, pH 7.3, 285 mOsm. The data were recorded with a Multiclamp 700B (Molecular Devices), digitized at 10 kHz, filtered at 2 kHz, collected when the series resistance fluctuated within 20% of the initial values and analyzed using pClamp 10.2 software (v.10.6.2.2., Molecular Devices). A fixed length of traces (2 min) was analyzed for the frequency and amplitude distributions of sEPSCs and sIPSCs using the MiniAnalysis (v6.03, Synaptosoft Inc.) program. In all experiments, series resistance was controlled below 20 MΩ and not compensated. Cells were rejected if the resting membrane potentials were more positive than −60 mV; or if series resistance fluctuated more than 20% of initial values.

### Immunofluorescence analysis

Animals were deeply anesthetized with 1% pentobarbital sodium (Sigma-Aldrich, # P3761) and then fixed with 4% paraformaldehyde (PFA) in PBS. After dissection, the brains were postfixed overnight in 4% PFA in PBS at 4 °C and dehydrated in 30% sucrose in PBS. The tissues were subsequently embedded in O.C.T. Compound (Tissue-Tek, #4583) and cryosectioned (Leica, #CM1850-1-1) at a thickness of 20 µm. The tissue slides were stored at −80 °C or washed three times in PBS prior to staining. The sections were incubated with a S100β primary antibody (1:100, Abcam #ab52642) and then with a secondary antibody conjugated to Alexa 488. Vectashield mounting medium (Vector, # H-1200) was used to seal the tissue. The sections were imaged using a laser confocal microscope (Nikon C2).

### Cell counting

Five slices of the mPFC were obtained from each mouse, and four mice from each group were used. The number of S100β^+^, tdTomato^+^, or S100β^+^ + tdTomato^+^ cells were counted, and DAPI labeling was used to identify the cells. ImageJ 1.50i software was employed for cell counting. We calculated the specificity of Cre recombinase expression as tdTomato^+^/(S100β^+^ + tdTomato^+^) and the efficiency of Cre recombinase expression as S100β^+^/(S100β^+^ + tdTomato^+^). The density of astrocytes/neurons was calculated by dividing the total number of S100β^+^/NeuN^+^ cells by the area of the mPFC region outlined with Adobe Illstrator software.

### FACS-droplet digital PCR

Brain slices containing the mPFC were prepared using standard methods for the electrophysiology experiments described above. The slices were blocked in an AP5, CNQX, and TTX cocktail to prevent excitotoxic cell death and then treated with the Papain Dissociation System (Worthington) following the manufacturer’s instructions. Then, the cells were labeled by Anti-ACSA-2-PE (Miltenyi, #130-102-365; ACSA-2: astrocyte cell surface antigen-2, specifically expressed on astrocytes) and immediately sorted by Beckman MoFlo XDP Cell Sorter system using a 100 µm nozzle, a sheath pressure of 10 psi, and in the single-cell sorting mode. To exclude dead cells, DAPI (DAPI*2HCl, Life Technologies Cat#D1306) was added to the single-cell suspension to the final concentration of 2 ng ml^−1^. ACSA + populations were chosen to select cells with low DAPI and high PE fluorescence. The sorted ACSA^+^ or ACSA^−^ cells were enriched by centrifugation (1000 × *g* for 3 min). After FACS, total RNA from the sorted cells was extracted with a RNeasy Micro Kit (QIAGEN, #74004), and the RNA concentration was assessed using a NanoDrop-1000. For droplet digital PCR, total RNA was reverse transcribed and amplified by a Discover-sc WTA Kit V2 (Vazyme, V2 N711-03) and a PrimeScriptTM RT reagent Kit (Takara, #RR037A). Droplet digital PCR was performed with QX200™ ddPCREvaGreenSupermix (Bio-Rad, #186-4033), and 18S RNA served as an internal control. PCR was performed on a QX200™ Droplet Digital™ PCR System (Bio-Rad, CA). The analysis was performed using QuantaSoft software (Bio-Rad, v 1.7.4.0917). The IP3R2 and 18S mRNA levels were examined with the following primers: IP3R2 forward, TGGTGGATGACCGTTGTG and reverse, GTATTGCTTCTGGGCAGAGTAT; 18 s forward, AGTTCCAGCACATTTTGCGAG and reverse, TCATCCTCCGTGAGTTCTCCA.

### Ca^2+^ imaging and fluorescence imaging of GRAB_ATP1.0_ sensors in brain slices

Coronal mPFC slices (300 μm) were made from 10- to 11-week-old male IP3R2 WT and IP3R2 KO mice. Animals were anesthetized with pentobarbital, the brain was removed swiftly and gently, then acute brain slices containing mPFC region were prepared in ice-cold oxygenated slicing buffer containing (in mM): 195 mM sucrose, 2 mM KCl, 0.2 mM CaCl_2_, 12 mM MgSO_4_, 1.3 mM NaH_2_PO_4_, 26 mM NaHCO_3_, and 10 mM glucose at the 0.14 mm/s cutting speed. Slices were then incubated at 34 °C in oxygenated ACSF containing (in mM): 126 mM NaCl, 3.0 mM KCl, 1.25 mM NaH_2_PO_4_, 2.0 mM CaCl_2_, 1.0 mM MgSO_4_, 26 mM NaHCO_3_, and 10 mM glucose for 30 min and then transferred at room temperature before experiments.

Astrocytes both expressing hM3Dq and GCaMP6m or hM3Dq and ATP1.0 were selected for imaging. An Olympus FV1200MPE two-photon microscope (Olympus FV1200MPE, Japan) equipped with a 25X, 1.05 NA water-immersion objective (Olympus, Japan) was used for imaging the slices. mCherry, GcaMP6m and ATP1.0 were exited at 920 nm, with a Mai Tai Ti:Sapphire laser. The imaging speed was set at 0.129 s/frame with 256 × 256 pixels in each frame. A glass pipette was guided at white light and placed near a target astrocyte. The pipette solution contained either ACSF or ACSF with CNO (10 mM) or ACSF with ATP (2 μM). Application of a 0.14 MPa increase in pressure to the pipette for 200 ms, resulted in an increase of the spread of solution and the fluorescence of the Ca^2+^ indicator or ATP sensor were measured from 60 s before to 60 s after drug application.

Time-lapse images of the brain slices were analyzed using Fiji to acquire the fluorescence intensity in the region of interest (ROI) in each frame. The change in fluorescence intensity (Δ*F*/*F*) as 100*(*F*_t_−*F*_0_)/*F*_0_, where *F*_t_ was the fluorescence intensity at time *t* and *F*_0_ was the average fluorescence intensity before the drug application.

### Primary astrocyte culture and ATP assessment

PFC astrocytes were obtained from 1- to 2-day-old IP3R2 KO mice, plated on 6-well cell culture dishes with astrocyte-specific medium (DMEM/F12 containing 10% FBS and 1% penicillin-streptomycin) and allowed to adhere for 24 h. After that, the astrocyte-specific-medium was refreshed to remove the non-adherent cells. Adherent astrocytes were maintained in a humidified incubator with 5% CO_2_ at 37 °C with medium change every 2 days. For ARL67156 treatment, the culture medium was changed to ARL67156-contained astrocyte-specific medium to inhibit the ATP hydrolyzation on Day 8. The extracellular and intracellular ATP assessment were carried out with CellTiter-Glo Reagent in Day 10. The cultured medium was collected to detect the extracellular ATP, while the adherent cells were harvested with strong RIPA containing ARL67156 and agitated organelles by sonication to estimate endogenous ATP and total protein concentration. These treated samples were mixed with equal the CellTiter-Glo Reagent in opaque-walled 96-well plates suitable for luminescence measurements and reacted for 12 min at room temperature. The ATP concentration was generated according to the standard curve of ATP disodium salt from 1 µM to 10 nM. The total protein concentration was assessed with BCA reagent to determine the relative quantity of ATP for each astrocyte.

### Statistical analyses

All data are presented as the mean ± SEM. The statistical analysis was conducted by SPSS 13.0 software. One-way ANOVA was used for multiple group comparisons. Differences between two groups were determined with Student’s *t*-test or Mann–Whitney U-test depending on its measure and distribution. All statistical tests were two-tailed, and significance was assigned at *P* < 0.05.

### Reporting summary

Further information on research design is available in the Nature Research Reporting Summary linked to this article.

## Supplementary information

Supplementary Movie 1

Supplementary Movie 2

Supplementary Movie 3

Supplementary Movie 4

Description of Additional Supplementary Files

Supplementary Information

Reporting Summary

## Data Availability

All data supporting the findings of this study are provided within the paper and its supplementary information. All additional information will be made available upon reasonable request to the authors. Source data are provided with this paper.

## Source data

Source Data
