# Supplementary material for: Impaired calcium signaling in astrocytes modulates autism spectrum disorder-like behaviors in mice
Source: Nat Commun. 2021 May 31;12:3321. doi: 10.1038/s41467-021-23843-0 (PMC8166865; doi:10.1038/s41467-021-23843-0)
Supplement: Supplementary file 5 — Description of Additional Supplementary Files [file 41467_2021_23843_MOESM5_ESM.docx]

**Supplementary Videos**

**Supplementary Video 1 ATP1.0 WT Gq+ACSF-representative movie for ATP fluctuations in mPFC hM3Dq-expressing astrocytes from IP3R2 WT mice treated with ACSF.**

**Supplementary Video 2 ATP1.0 KO Gq+ACSF-representative movie for ATP fluctuations in mPFC hM3Dq-expressing astrocytes from IP3R2 KO mice treated with ACSF.**

**Supplementary Video 3 ATP1.0 WT Gq+CNO-representative movie for ATP fluctuations in mPFC hM3Dq-expressing astrocytes from IP3R2 WT mice treated with CNO.**

**Supplementary Video 4 ATP1.0 KO Gq+CNO-representative movie for ATP fluctuations in mPFC hM3Dq-expressing astrocytes from IP3R2 KO mice treated with CNO.**
