# Supplementary material for: Impaired calcium signaling in astrocytes modulates autism spectrum disorder-like behaviors in mice
Source: Nat Commun. 2021 May 31;12:3321. doi: 10.1038/s41467-021-23843-0 (PMC8166865; doi:10.1038/s41467-021-23843-0)
Supplement: Supplementary file 6 — Supplementary Information [file 41467_2021_23843_MOESM6_ESM.pdf]

Supplementary Information

Impaired calcium signaling in astrocytes modulates autism spectrum disorder-like behaviors in mice

Qian Wang, Ying Kong, Ding-Yu Wu, Ji-Hong Liu, Wei Jie, Qiang-Long You, Lang Huang, Jian Hu, Huai-De Chu, Feng Gao, Neng-Yuan Hu, Zhou-Cai Luo, Xiao-Wen Li, Shu-Ji Li, Zhao-Fa Wu, Yu-Long Li, Jian-Ming Yang and Tian-Ming Gao

Supplementary Figure 1

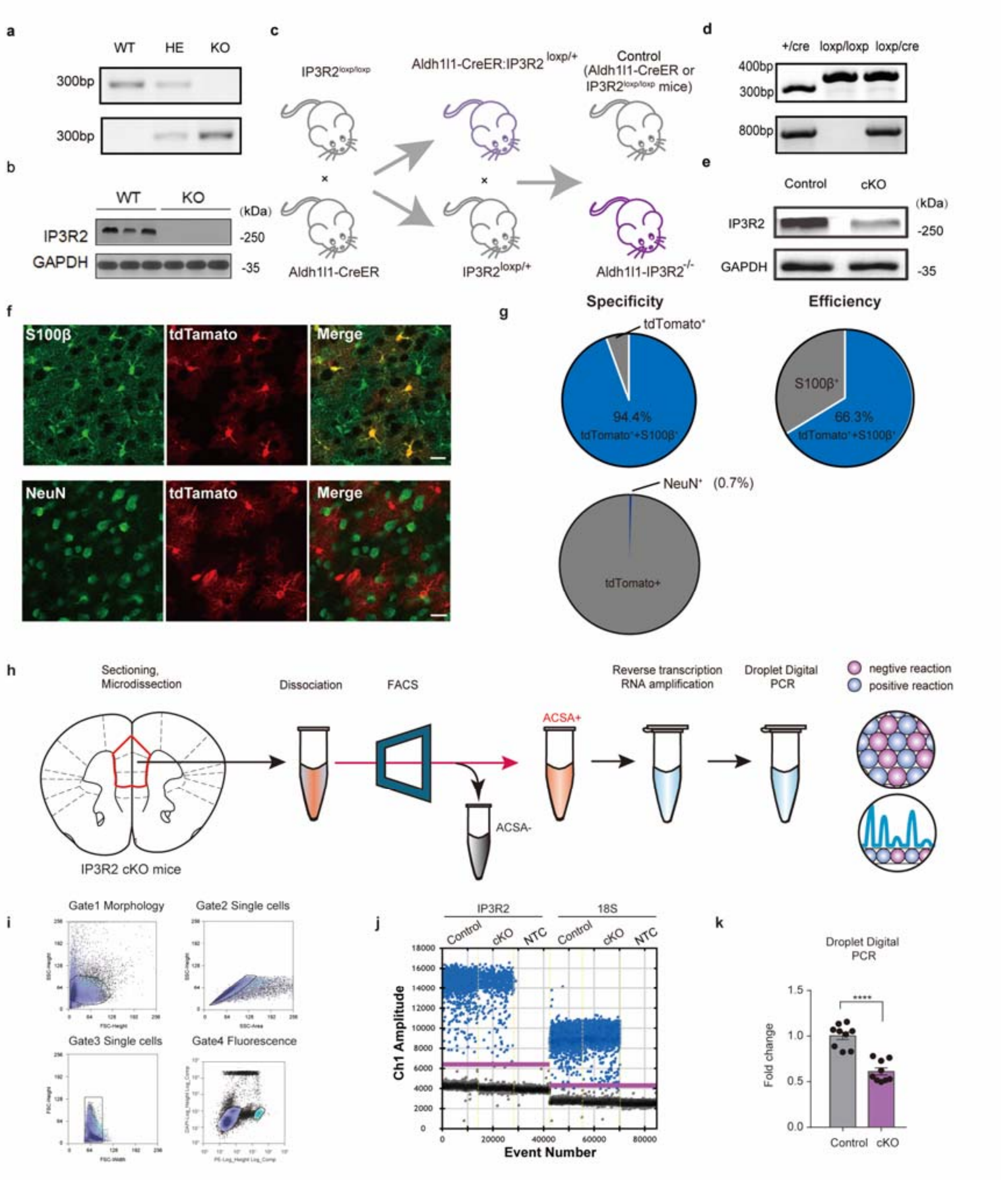

Supplementary Figure 1 Identification of IP3R2 expression in IP3R2 mutant mice by PCR and Western blot analyses.

a, Genotyping results of WT and IP3R2 KO mice by tail DNA PCR. **b,** Western blot analysis of WT and IP3R2 KO mice. **c,** Generation of IP3R2 cKO mice. **d,** Genotyping results of control and IP3R2cKO mice. **e,** Western blot analysis of control

and IP3R2 cKO mice. **f**, tdTomato (Ai14 reporter) overlapping with S100β (an astrocyte-specific marker) or NeuN (a neuron-specific marker) in the mPFC of Aldh1l1-CreER:Ai14 mice after injection with tamoxifen and staining for S100β or NeuN (green). Scale bar: 20 μm. **g**, Statistical graphs showing the specificity (left, the percentage of tdTomato-positive cells that expressed S100β, 94.44 ± 0.70%, $n = 4$, total number of cells: 791) and efficiency (right, the percentage of S100β-positive cells that expressed tdTomato, 66.30 ± 1.77%, $n = 4$) of Cre-mediated recombination in the mPFC in Aldh1l1-CreER:Ai14 mice. Only ~0.7% of tdTomato-positive cells expressed NeuN in the mPFC in Aldh1l1-CreER:Ai14 mice. Total number of cells: 663, $n = 4$ mice. **h**, A schematic diagram showing the procedure for FACS-droplet digital PCR. **i**, Plots showing the purification of astrocytes by FACS. $ACSA^+$ and $ACSA^-$ (ACSA-2: astrocyte cell surface antigen-2, a general astrocyte marker) cells were FACS-sorted. **j**, A one-dimensional (1-D) plot data showing IP3R2 mRNA in $ACSA^+$ and $ACSA^-$ cells, as determined by droplet digital PCR. The blue and black plots denote positive and negative signals, respectively. **k**, A statistical graph showing a reduction in the expression of the IP3R2 gene in cKO astrocytes compared to control astrocytes (two-tailed unpaired t test. $t_4 = 6.887, P = 0.4*10^{-5}, n = 9$). The data are presented as the mean ± SEM. ****P < 0.0001. **a, d** repeated for 4 times; **b, d** repeated for 3 times.

Supplementary Figure 2

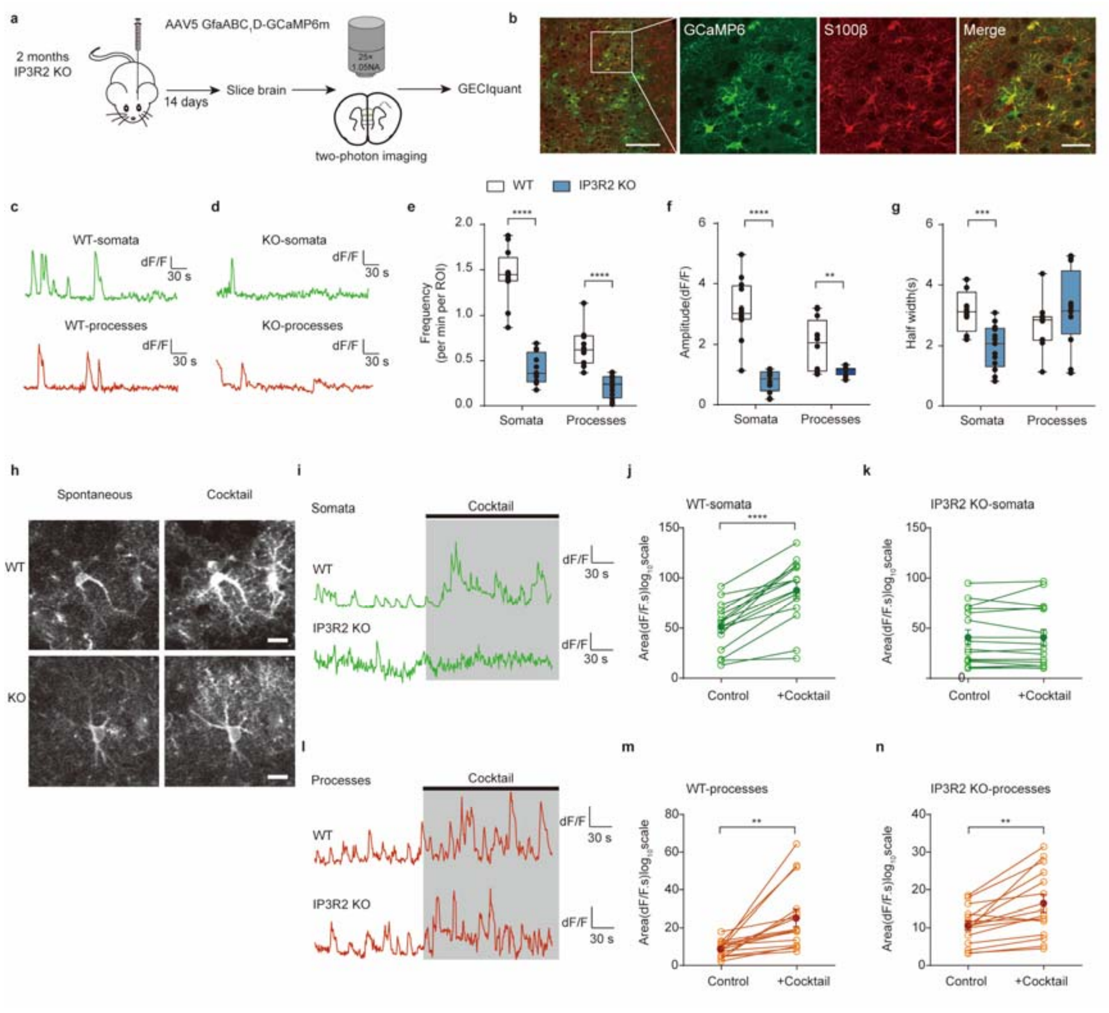

Supplementary Figure 2 Ca^{2+} signals in mPFC astrocytes from IP3R2 WT and KO mice.

a, Schematic illustrating the experimental approach. **b,** GCaMP6m (green) was expressed in astrocytes (astrocytic marker S100β, red). Scale bars: 200 µm, 50 µm. **c,** Representative images and traces for Ca^{2+} signals measured in the somata (green) and processes (red) of an astrocyte from an IP3R2 WT mouse. **d,** Same as **c**, but for an astrocyte from an IP3R2 KO mouse. **e-g,** Average data for Ca^{2+} fluctuation properties in IP3R2 WT and KO mice. (Frequency, *somata: WT vs. KO*, t_{22} *=10.65*, *P = 0*, *processes: WT vs. KO*, t_{22}*= 6.102*, $P=0.4*10^{-5}$; Amplitude, *somata: WT vs. KO*,

t_{22}=*7.862,P = 0.2*10^{-5}*, processes: WT vs. KO,* t_{22} *= 3.390, P = 0.0026*; Half width, *somata: WT vs. KO,* t_{23} *= 4.223, P = 0.0003, processes: WT vs. KO,* t_{22} *= 1.198, P = 0.2438*; *somata* : n = 82 and 7 events， *processes*: n = 583 and 207 events, The numbers refer to the numbers of Ca^{2+} fluctuations for the WT and IP3R2 KO mice.12 astrocytes from 5 mice for each genotype). Box plots present, in ascending order, minimum sample value, first quartile, median, third quartile, and maximum sample value. **h**, Representative images for GPCR-evoked Ca^{2+} signals in astrocytes from IP3R2 WT and KO mice. Scale bar:10μm. **i-k,** Representative traces and average data for GPCR-evoked Ca^{2+} signals in astrocytic somata from IP3R2 WT and KO mice. (Cocktail: 500 μM ATP, 50 μM DHPG, 10 μM Carbachol. **j**: WT-somata: *Control vs. Cocktail,* t_{14} *= 7.281, P=0.4*10^{-5}*; **k**: KO-somata: *Control vs. Cocktail,* t_{14} *= 0.05873, P = 0.9540*). **l-n,** Same as **i**–**k**, but for astrocytic processes (**m**: WT-processes: *Control vs. Cocktail,* t_{14}*= 3.723, P = 0.0023*; **n**: KO-processes: *Control vs. Cocktail,* t_{14} *= 3.826, P = 0.0019*). n = 15 astrocytes from 5 mice for each genotype. WT, wild-type mice; KO, IP3R2 null mutant mice. The data are presented as the mean ± SEM. Two-tailed unpaired t test (**e-g**). Two-tailed paired t test (**j-k; m-n**). **P < 0.01, ***P < 0.001, ****P < 0.0001. Comparisons with no asterisk had a P > 0.05 and were considered not significant.

Supplementary Figure 3

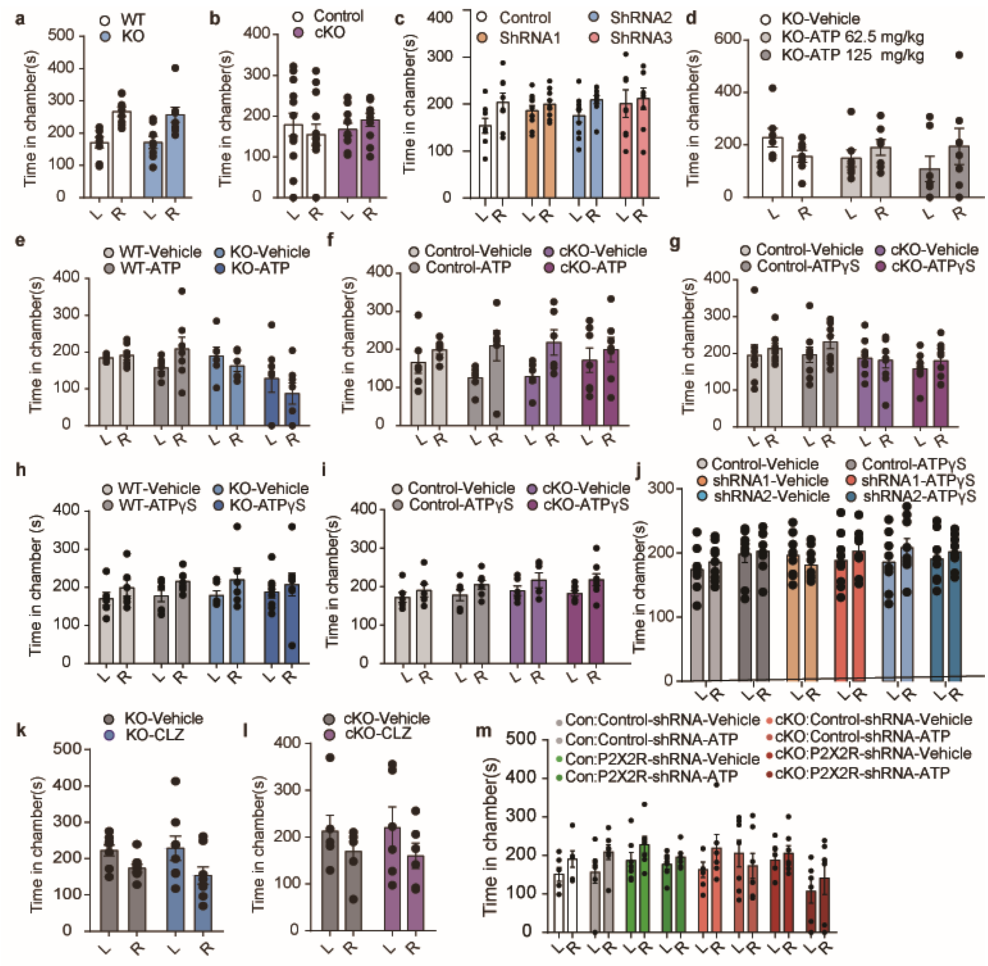

Supplementary Figure 3 No preference for the left or right chamber in the three-chamber test.

a, WT and IP3R2 KO mice equally explored the two opposite chambers (L: left and R: right) during the first 10-min habituation phase, suggesting no pre-existing chamber bias. Two-tailed unpaired t test, WT_L vs. WT_R, $t_{14} = 2.121$, $P = 0.0523$, $n = 8$; KO_L vs. KO_R, $t_{14} = 0.4934$, $P = 0.6294$, $n = 8$). **b,** Same as **a**, but for control and IP3R2 cKO mice. Two-tailed unpaired t test, control $_L$ vs. control $_R$, $t_{20} = 1.057$, $P = 0.3031$, $n = 11$; cKO $_R$ vs. cKO $_L$, $t_{18} = 1.043$, $P = 0.3109$, $n = 10$. **c**, ATP treatment did not affect the left or right preference of C57BL/6J mice injected with AAV carrying GFAP-IP3R2

shRNA or control-shRNA. (Control $_L$vs Control $_R$, t_{14} = 1.993, P = 0.0661, n = 8; shRNA1 $_L$ vs shRNA1 $_R$, t_{18} = 0.9535, P = 0.353, n = 10; shRNA2 $_L$ vs shRNA2 $_R$, t_{18}= 2.079, P = 0.0522, n = 10; shRNA3 $_L$ vs shRNA3 $_R$, t_{14} = 0.2845, P = 0.7802, n = 8). **d,** Statistical data for IP3R2 KO mice that received vehicle or ATP treatment. Two-tailed unpaired t test, KO-vehicle $_L$ vs. KO-vehicle $_R$, t_{12} = 0.1110, P = 1.721, n = 7; KO-62.5 $_L$ vs. KO-62.5 $_R$, t_{12} = 0.9304, P = 0.3705, n = 7; KO-125 $_L$ vs. KO-125 $_R$, t_{12} = 1.018, P = 0.3289, n = 7. **e,** Statistical data for WT and IP3R2 KO mice that received vehicle or ATP treatment. WT-vehicle $_L$ vs. WT-vehicle $_R$, Mann-Whitney U test, U = 23, P = 0.9015, n = 7; WT-ATP $_L$ vs. WT-ATP $_R$, Mann-Whitney U test, U = 13, P = 0.1649, n = 7; KO-vehicle $_L$ vs. KO-vehicle $_R$, two-tailed unpaired t test, t_{10} = 0.9030, P = 0.3878, n = 6; KO-ATP $_L$ vs. KO-ATP $_R$, two-tailed unpaired t test, t_{10} = 1.01, P = 0.3362, n = 7. **f,** Same as **e**, but for control and IP3R2 cKO mice. Control-vehicle $_L$ vs. control-vehicle $_R$, two-tailed unpaired t test, t_{10} = 1.062, P = 0.3133, n = 6; control-ATP $_L$ vs. control-ATP $_R$, Mann-Whitney U test, U = 6, P = 0.0649, n = 6; cKO-vehicle $_L$ vs. cKO-vehicle $_R$, Mann-Whitney U test, U = 8, P = 0.1275, n = 6; cKO-ATP $_L$ vs. cKO-ATP $_R$, two-tailed unpaired t test, t_{12} = 0.6247, P = 0.5439, n = 7. **g,** Same as **f**, but for vehicle or ATPγS treatment. Two-tailed unpaired t test, control-vehicle $_L$ vs. control-vehicle $_R$, t_{14} = 0.5736, P = 0.5754, n = 8; control-ATPγS $_L$ vs. control-ATPγS $_R$, t_{16} = 1.261, P = 0.2254, n = 9; cKO-vehicle $_L$ vs. cKO-vehicle $_R$, t_{16} = 0.2296, P = 0.8213, n = 9; cKO-ATPγS $_L$ vs. cKO-ATPγS $_R$, t_{16} = 1.027, P = 0.3198, n = 9. **h**: Same as **e**, but for intra-mPFC injection of ATPγS. Two-tailed unpaired t test, WT-vehicle $_L$ vs. WT-vehicle $_R$, t10 = 1.068, P = 0.6259, n

= 6; WT-ATPγS $_L$ vs. WT-ATPγS $_R$, t_{12} = 2.102, P = 0.0574, n = 7; KO-vehicle $_L$ vs. KO-vehicle $_R$, t_{10} = 1.243, P = 0.2422, n = 6; KO-ATPγS $_L$ vs. KO-ATPγS $_R$, t_{14} = 0.5658, P = 0.5805, n =8. **i**: Same as **g**, but for intra-mPFC injection. Two-tailed unpaired t test, control-vehicle $_L$ vs. control-vehicle $_R$, t_{10} = 0.8282, P = 0.4269, n = 6; control-ATPγS $_L$ vs. control-ATPγS $_R$, t_{12} = 1.540, P = 0.1494, n = 7; cKO-vehicle L vs. cKO-vehicle $_R$, t_{10} = 1.191, P = 0.2613, n = 6; cKO-ATPγS $_L$ vs. cKO-ATPγS $_R$, t_{14} = 2.088, P = 0.0556, n = 8. **j,** Same as **i**, but for AAV-GFAP-IP3R2 shRNA mice. Two-tailed unpaired t test, control-vehicle $_L$ vs. control-vehicle $_R$, t_{16} = 0.8244, P = 0.4218, n = 9; control-ATPγS $_L$ vs. control-ATPγS $_R$, t_{16} = 0.2854, P = 0.7790, n = 9; shRNA1-vehicle L vs. shRNA1-vehicle $_R$, t_{16} = 1.082, P = 0.2952, n = 9; shRNA1-ATPγS $_L$ vs. shRNA1-ATPγS $_R$, t_{16} = 0.8150, P = 0.4271, n = 9. shRNA2-vehicle L vs. shRNA2-vehicle $_R$, t_{16} = 1.108, P = 0.2841, n = 9; shRNA2-ATPγS $_L$ vs. shRNA2-ATPγS $_R$, t_{16} = 0.7238, P = 0.4796, n = 9. **k,** Statistical data for IP3R2 KO mice injected with vehicle or clonazepam (CLZ). Two-tailed unpaired t test, KO-vehicle $_L$ vs. KO-vehicle $_R$, t_{14} = 1.581, P = 0.1361, n = 8; KO-CLZ $_L$ vs. KO-CLZ $_R$, t_{14} = 1.822, P = 0.0898, n = 8. **l,** Same as **g**, but for IP3R2 cKO mice. Two-tailed unpaired t test, cKO-vehicle $_L$ vs. cKO-vehicle $_R$, t_{10} = 1.075, P = 0.3076, n = 6; cKO-CLZ $_L$ vs. cKO-CLZ $_R$, t_{10} = 1.151, P = 0.2766, n = 6. **m,** ATP treatment did not affect the left or right preference of control mice or IP3R2 cKO mice injected with AAV carrying P2X2R-shRNA or control-shRNA. Two-tailed unpaired t test, Con:Control-vehicle$_L$ vs Con: Control-vehicle $_R$, t_{10} = 1.430, P = 0.1833, n = 6; Con:Control-ATP$_L$ vs Con:Control-ATP$_R$, t_{10} = 1.527, P = 0.1527, n =

7; Con:P2X2R-shRNA-vehicle $_{L}$ vs Con:P2X2R-shRNA-vehicle $_{R}$, $t_{12} = 1.366$, $P = 0.1968$, $n = 7$; Con: P2X2R-shRNA-ATP $_{L}$ vs Con: P2X2R-shRNA-ATP $_{R}$, $t_{12} = 1.156$, $P = 0.2703$, $n = 7$; cKO:Control-shRNA-vehicle$_{L}$ vs cKO:Control-shRNA-vehicle$_{R}$, $t_{10} = 1.362$, $P = 0.2029$, $n = 6$; cKO:Control-shRNA-ATP$_{L}$ *vs* cKO:Control-shRNA-ATP$_{R}$, $t_{12} = 0.6826$, $P = 0.5078$, $n = 7$; cKO:P2X2R-shRNA-vehicle $_{L}$ vs cKO:P2X2R-shRNA-vehicle $_{R}$, $t_{12} = 0.6595$, $P = 0.5220$, $n = 7$; cKO:P2X2R-shRNA-ATP $_{L}$ vs cKO:P2X2R-shRNA-ATP $_{R}$, $t_{12} = 1.151$, $P = 0.2720$, $n = 7$. The data are presented as the mean ± SEM. Comparisons with no asterisk had a $P > 0.05$ and were considered not significant.

Supplementary Figure 4

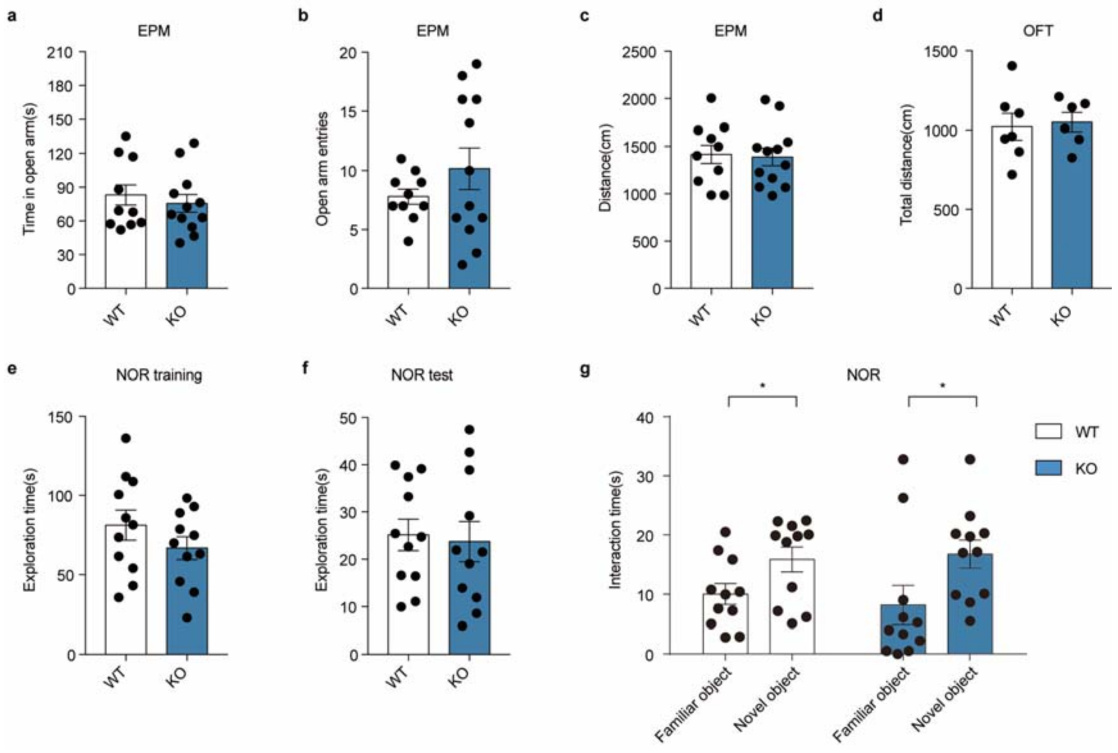

Supplementary Figure 4 IP3R2 KO mice show enhanced learning and memory and normal anxiety-like behaviors and locomotor activity.

a-c, The performance of WT and IP3R2 KO mice in the elevated plus-maze (EPM) test. **a,** The time spent in the open arms (t_{20} = 0.5367, P = 0.5974, n = 10/12), **b,** The number of entries into the open arms (t_{20} = 1.166, P = 0.2572, n = 10/12). **c,** Traveled distance (t_{20} = 0.2268, P = 0.8229, n = 10/12)**. d**, The total distance traveled by WT and IP3R2 KO mice over 5 min in the open field test (OFT) (t_{11} = 0.27, P = 0.7922, n = 7/6). **e-f**, The total time spent exploring the objects in the novel object recognition (NOR) test. **e**, Training session (t_{20} = 1.209, P = 0.2408, n = 11). **f**, Test session (t_{20} = 0.2600, P = 0.7975, n =11). **g**, The total time that WT and IP3R2 KO mice spent exploring the familiar and novel objects in the NOR test session (t_{WT} =2.129, P = 0.0459, n =11; t_{KO} =2.106, P = 0.0480, n =11). The data are presented as the mean ± SEM. *P < 0.05. Two-tailed unpaired t test. Comparisons with no asterisk had a P > 0.05 and were considered not significant.

Supplementary Figure 5

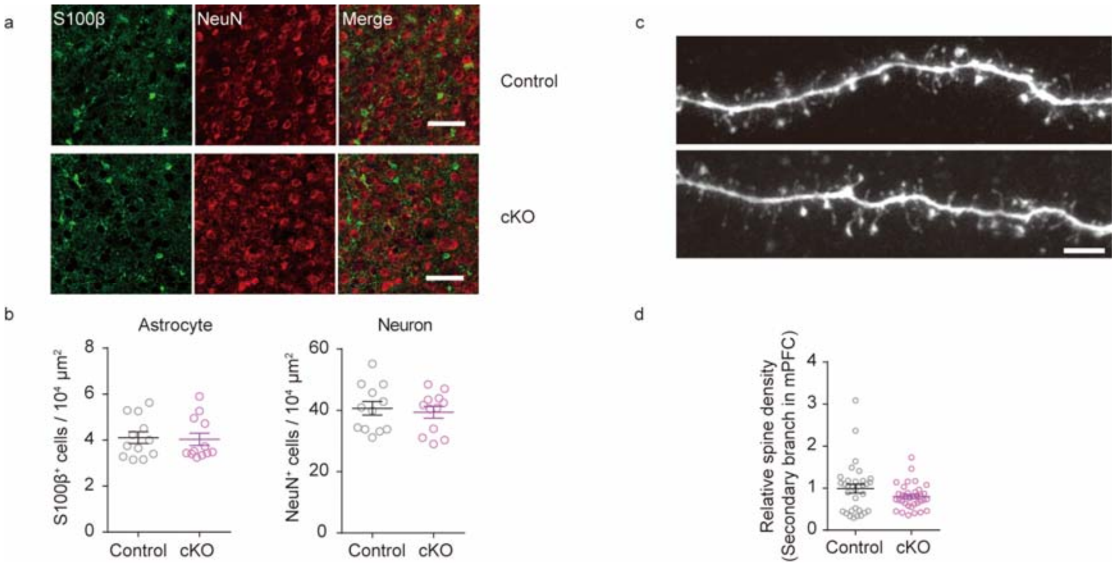

Supplementary Figure 5 No alterations of astrocyte and neuronal morphology and density were observed in the mPFC of IP3R2 cKO mice.

a, Representative images of sections immunolabeled with the astrocyte marker S100β (green) and neuronal marker NeuN (red) from Control and IP3R2 cKO mice in the mPFC. Scale bar, 20 μm. **b**, Quantification of the density of astrocytes and neurons in the mPFC from Control and IP3R2 cKO mice (astrocyte: $t_{22} = 0.2013$, $P = 0.8423$, $n = 12$; neuron: $t_{22} = 0.4388$, $P = 0.6651$, $n = 12$, Two-tailed unpaired t test). **c**, Representative confocal images of biocytin-stained secondary dendrites of mPFC neurons from IP3R2 cKO and Control mice. Scale bar; 5 μm. **d**, Quantification of spine density of mPFC dendrites from IP3R2 cKO and Control mice ($U = 461$, $P = 0.1549$, n =33/35, from 5/6 mice; Mann Whitney test). Control, Aldh1L1-CreER mice; cKO, IP3R2 cKO mice. The data are presented as the mean ± SEM. Comparisons with no asterisk had a $P > 0.05$ and were considered not significant.

Supplementary Figure 6

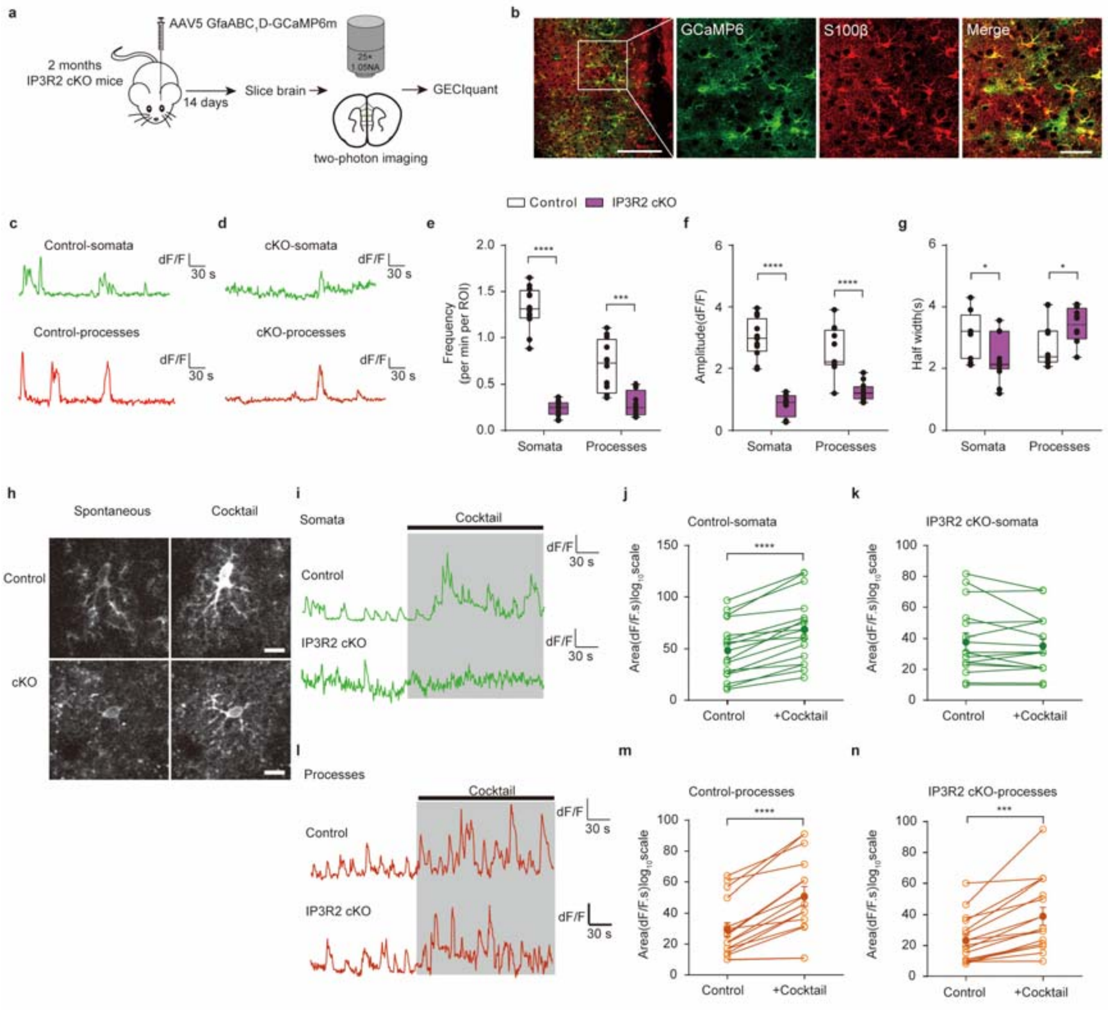

Supplementary Figure 6 Ca^{2+} signals in mPFC astrocytes from IP3R2 Control and cKO mice.

a, Schematic illustrating the experimental approach. **b,** GCaMP6m (green) was expressed in astrocytes (astrocytic marker S100β, red). Scale bars: 200 μm, 50 μm. **c,** Representative traces for Ca^{2+} signals measured in the soma (green) and processes (red) of an astrocyte from an IP3R2 Control mouse. **d,** Same as **c**, but for an astrocyte from an IP3R2 cKO mouse. **e-g,** Average data for Ca^{2+} fluctuation properties in IP3R2 Control and cKO mice. (Frequency, *somata: Control vs. cKO*, $t_{22} = 15.42$, $P = 0$, *processes: Control vs. cKO*, $t_{22} = 4.633$, $P = 0.0001$; Amplitude, *somata: Control vs.*

cKO, t_{22} *= 10.22, P = 0*, *processes: Control vs. cKO*, t_{22} *= 5.636*, *P = 0.58*$*10^{-4}$; Half width, *somata: Control vs. cKO*, t_{22} *=2.505*, *P = 0.0198*, *processes: Control vs. cKO,* t_{22} *= 2.390, P = 0.0258*; *somata*：n= 87 and 10 events, *processes*: n= 551 and 182 events. The numbers refer to the numbers of Ca^{2+} fluctuations for the WT and IP3R2 cKO mice. 12 astrocytes from 5 mice for each genotype). Box plots present, in ascending order, minimum sample value, first quartile, median, third quartile, and maximum sample value. **h**, Representative images for GPCR-evoked Ca^{2+} signals in astrocytes from IP3R2 Control and cKO mice. Scale bar: 10μm. **i-k,** Representative traces and average data for GPCR-evoked Ca^{2+} signals in astrocytic somata from IP3R2 Control and cKO mice. (Cocktail: 500 μM ATP,50 μM DHPG,10 μM Carbachol; **j**: Control-somata: *Control vs. Cocktail,* t_{15} *= 6.995*, *P = 0.4*$*10^{-5}$; **k**: cKO-somata: *Control vs. Cocktail,* t_{15} *= 1.142*, *P = 0.2712*). **l-n,** Same as **i**–**k**, but for astrocytic processes (**m**: Control-processes: *Control vs. Cocktail,* t_{15} *= 6.968*, *P =0.5*$*10^{-5}$; **n**: cKO-processes: *Control vs. Cocktail,* t_{14} *= 4.969*, *P = 0.0002*). *n* = 16 cells from 5 mice for each genotype. Control, IP3R2$^{loxp/loxp}$ mice; cKO, IP3R2 cKO mice. The data are presented as the mean ± SEM. Two-tailed unpaired t test (**e-g**). Two-tailed paired t test (**j-k; m-n**). *P<0.05, ***P<0.001, ****P<0.0001. Comparisons with no asterisk had a P > 0.05 and were considered not significant.

Supplementary Figure 7

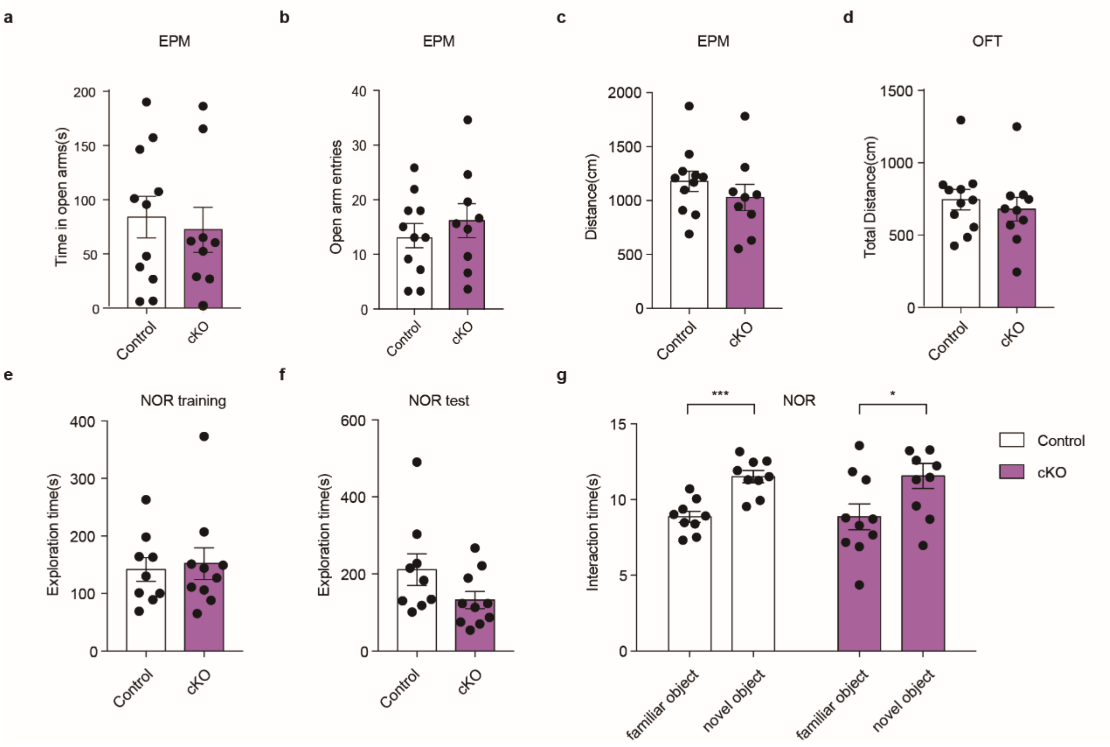

Supplementary Figure 7 IP3R2 cKO mice show normal learning and memory, anxiety-like behaviors and locomotor activity.

a-c, The performance of control and IP3R2 cKO mice in the EPM test. **a,** The time spent in the open arms (t_{18} = 0.414, P = 0.6838, n = 9/11), **b,** The number of entries into the open arms (t_{18} = 0.8445, P= 0.4095, n = 9/11). **c,** The distance traveled (t_{18} = 0.9917, P = 0.3345, n = 9/11)**. d**, The total distance traveled by control and IP3R2 cKO mice over 5 min in the OFT (t_{19} = 0.6179, P = 0.5440, n = 10/11). **e-f**, The total time spent exploring the objects in the NOR test. **e**, Training session (t_{17} = 0.292, P = 0.7738, n = 9/10). **f**, Test session (t_{17} = 1.742, P = 0.0996, n = 9/10). **g**, The total time that control and IP3R2 cKO mice spent exploring the familiar and novel objects in the NOR test session (Control: t_{16} = 4.911, P = 0.0002, n = 9; cKO: t_{18}= 2.263, P = 0.0362,

n = 10). The data are presented as the mean ± SEM. Two-tailed unpaired t test. *P < 0.05, ***P<0.001. Comparisons with no asterisk had a P > 0.05 and were considered not significant.

Supplementary Figure 8

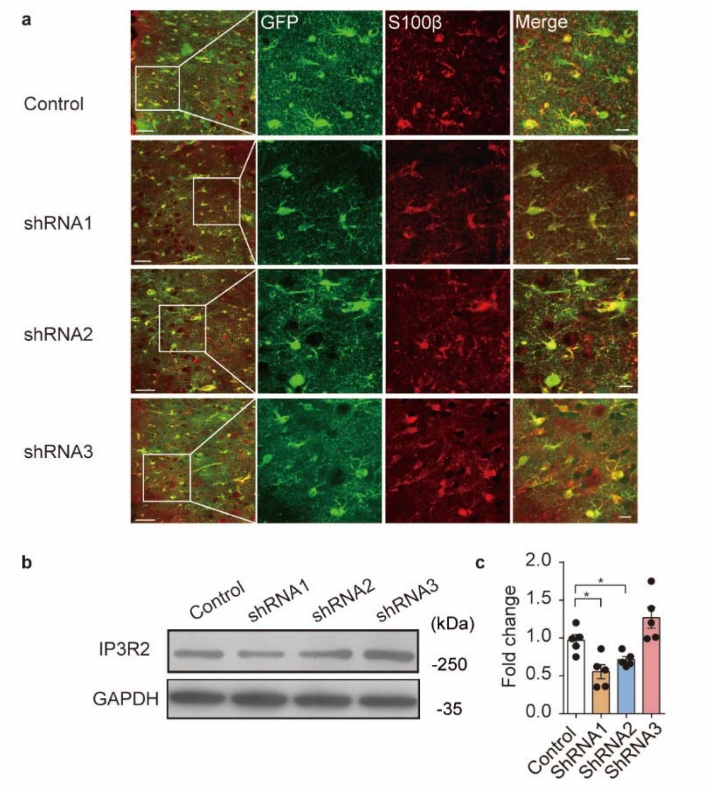

Supplementary Figure 8 The specificity of GFAP-IP3R2 shRNAs.

a, GFAP-IP3R2 shRNAs-expressing astrocytes were co-stained with the astrocytic marker S100β in the mPFC. Scale bars: 50 μm, 10 μm. **b-c,** Western blot showing the knockdown of IP3R2 in the mPFC of mice injected with the AAV vector carrying GFAP-IP3R2-shRNAs. (*Control-shRNA vs. IP3R2-shRNA1*, t_8= 3.3471, *P* =0.0101, *n* = 5; *Control-shRNA vs. IP3R2-shRNA2*, t_8= 3.004, *P* =0.0170, *n* = 5; *Control-shRNA vs. IP3R2-shRNA3*, t_8= 1.1883, *P*=0.0964, *n* = 5). The data are presented as the mean

± SEM. Two-tailed unpaired t test. *P < 0.05. Comparisons with no asterisk had a P > 0.05 and were considered not significant.

Supplementary Figure 9

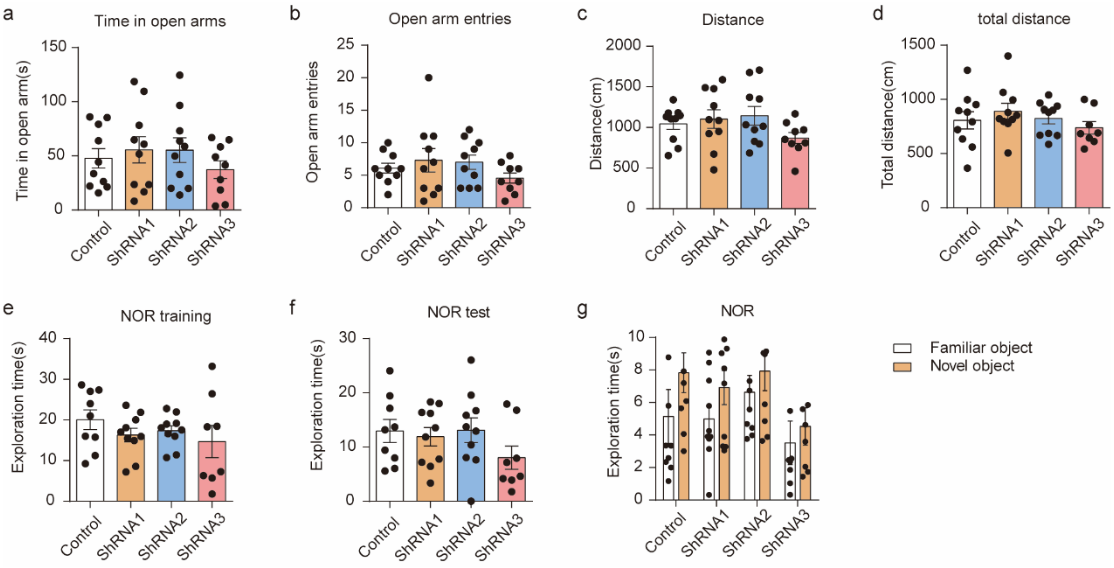

Supplementary Figure 9 Mice with astrocytic IP3R2 specifically knockdown in the mPFC show normal learning and memory, anxiety-like behaviors and locomotor activity.

a-c, The performance of C57BL/6J mice that received GFAP-shRNA AAV in the EPM test. **a,** The time spent in the open arms (Control vs. shRNA1, t_{18} = 0.5162, *P* = 0.612, *n* = 10; Control vs. shRNA2, t_{18} = 0.5202, *P* = 0.6092, *n* = 10; Control vs. shRNA3, t_{17} = 0.8590, *P* = 0.4023, *n* = 10/9), **b,** The number of entries into the open arms (*Control vs. shRNA1*, *U* = 46.5, *P* = 0.81, *n* = 10; *Control vs. shRNA2*, t_{18} = 0.6773, *P* = 0.5068, *n* = 10; *Control vs. shRNA3*, t_{17} = 1.42, *P* = 0.1734, *n* = 10/9). **c,** The distance traveled (*Control vs. shRNA1*, t_{18} =0.4368, *P* = 0.6674, *n* = 10; *Control vs. shRNA2*, t_{18} = 0.7395, *P*= 0.4691, *n* = 10; *Control vs. shRNA3*, t_{17} = 1.804, *P* =

0.089, n =10/9). **d**, The total distance traveled by C57BL/6J mice that received GFAP-shRNA AAV over 5 min in the OFT (*Control vs. shRNA1*, t_{18} = 0.7725, P =0.4499, n = 10; *Control vs. shRNA2*, t_{18} = 0.1816, P = 0.8579, n = 10; *Control vs. shRNA3*, t_{16} = 0.6681, P = 0.5136, n = 10/8). **e-f**, The total time spent exploring the objects in the NOR test. **e**, Training session (*Control vs. shRNA1*, t_{17} = 0.1.306, P = 0.2089, n = 9/10; *Control vs. shRNA2*, t_{17} = 1.026, P = 0.3193, n = 9/10; *Control vs. shRNA3*, t_{15} = 1.183, P = 0.255, n = 9/8). **f**, Test session (*Control vs. shRNA1*, t_{17} = 0.3885, P = 0.7024, n = 9/10; *Control vs. shRNA2*, t_{17} = 0.04497, P = 0.9547, n = 9/10; *Control vs. shRNA3*, t_{15} = 1.626, P = 0.1247, n = 9/8). **g**, The total time that control and IP3R2-knockdown mice spent exploring the familiar and novel objects in the NOR test (*Control vs. Control*, t_{16} = 1.309, P = 0.2092 n = 9; *shRNA1 vs. shRNA1*, t_{18} = 1.433, P = 0.169, n = 10; *shRNA2 vs. shRNA2*, t_{16} = 0.8215, P = 0.4234, n = 9; *shRNA3 vs. shRNA3*, t_{14} = 0.5730, P = 0.5757, n = 8). The data are presented as the mean ± SEM. Two-tailed unpaired t test. Comparisons with no asterisk had a P > 0.05 and were considered not significant.

Supplementary Figure 10

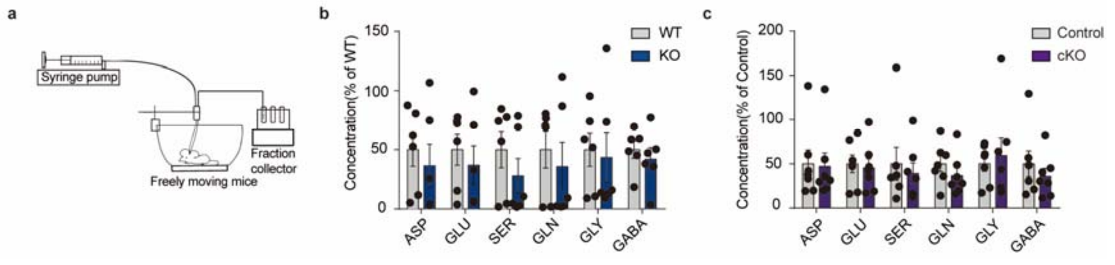

Supplementary Figure 10 No change in the levels of gliotransmitters in the mPFC of IP3R2 mutant mice.

a, A diagram of the *in vivo* microdialysis assay. **b**, The concentrations of aspartate (ASP: t_{10} = 0.5863, P = 0.5706, n = 6), glutamate (GLU; t_{10} = 0.6247, P = 0.5461, n = 6), serine (SER; t_{10} = 1.042, P = 0.3219, n = 6), glutamine (GLN; t_{10} = 0.5549, P = 0.5911, n = 6), glycine (GLY; t_{10} = 0.2587, P = 0.8011, n = 6), and GABA (t_{10} = 0.6514, P = 0.5295, n = 6) in the mPFC were not different between WT and IP3R2 KO mice. **c**, The concentrations of ASP (t_{12} = 0.1371, P = 0.8932, n = 7), GLU (t_{12} = 0.3281, P = 0.7485, n = 7), SER (t_{12} = 0.4886, P = 0.6339, n = 7), GLN (t_{12} = 1.073, P = 0.3044, n = 7), GLY (t_{12} = 0.423, P = 0.6798, n = 7) and GABA (t_{12} = 0.8277, P = 0.4240, n = 7) in the mPFC were not different between control and IP3R2 cKO mice. WT, wild-type mice; KO, IP3R2$^{-/-}$ mice; control, IP3R2$^{loxp/loxp}$ mice; cKO, IP3R2 cKO mice. The data are presented as the mean ± SEM. Two-tailed unpaired t test. Comparisons with no asterisk had a P > 0.05 and were considered not significant.

Supplementary Figure 11

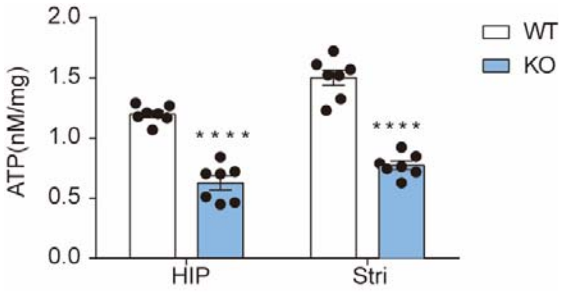

Supplementary Figure 11 The ATP levels in the hippocampus and striatum from IP3R2 WT and KO mice. ATP levels in the hippocampus: t_{12} =8.951, p=0.1*10^{-5}, n = 7; ATP levels in the striatum: t_{12} = 9.937, p= 0, n = 7. WT, wild-type mice; KO, IP3R2 null mutant mice. The data are presented as the mean ± SEM. Two-tailed unpaired t test was used. ****P<0.0001.

Supplementary Figure 12

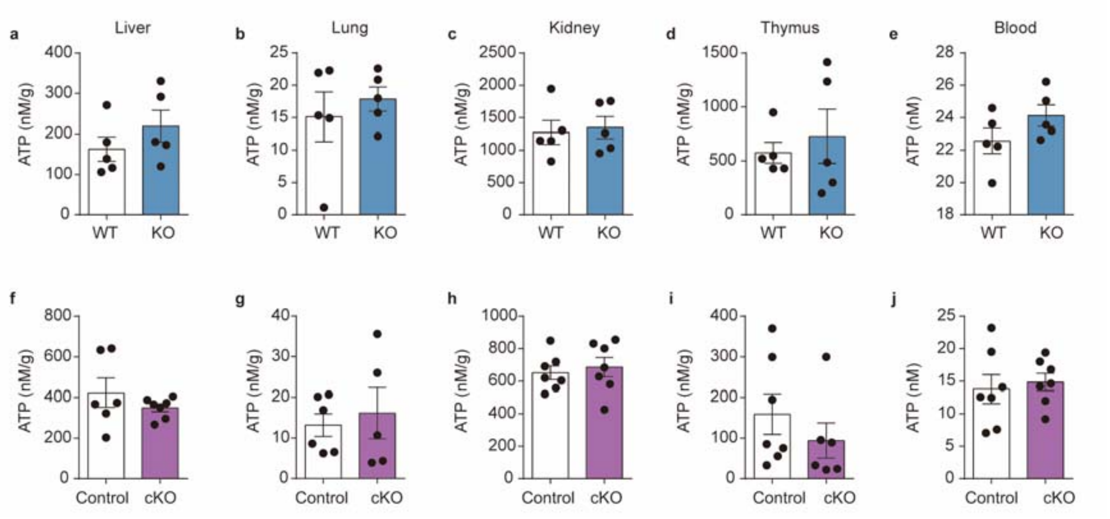

Supplementary Figure 12 The ATP levels in the peripheral organs of IP3R2 KO and IP3R2 cKO mice.

a-e, The ATP levels in the peripheral organs of IP3R2 KO mice. Liver, $U = 17$, $P = 0.6154$, $n = 6/7$; Lung, $U = 15$, $P = 0.9999$, $n = 6/5$; Kidney, $t_{12} = 0.4809$, $P = 0.6392$, $n = 7$; Thymus, $U = 15$, $P = 0.4312$, $n = 6/7$; Blood, $t_{12} = 0.4231$, $P = 0.6797$, $n = 7$. **f-j**, The ATP levels in the peripheral organs of IP3R2 cKO mice. Liver, $t_8 = 1.143$, $P = 0.2859$, $n = 5$; Lung, $t_8 = 0.6425$ $P = 0.5385$, $n = 5$; Kidney, $t_8 = 0.2942$, $P = 0.7761$, $n = 5$; Thymus, $t_8 = 0.5678$, $P = 0.5858$, $n = 5$; Blood, $t_8 = 1.530$, $P = 0.1644$, $n = 5$. WT, wild-type mice; KO, IP3R2 null mutant mice; control, Aldh1L1-CreER mice; cKO, IP3R2 cKO mice. The data are presented as the mean ± SEM. Two-tailed unpaired t test (**c**, **e**, **f-i**). Mann-Whitney U-test (**a**, **b**, **d**). Comparisons with no asterisk had a P > 0.05 and were considered not significant.

Supplementary Figure 13

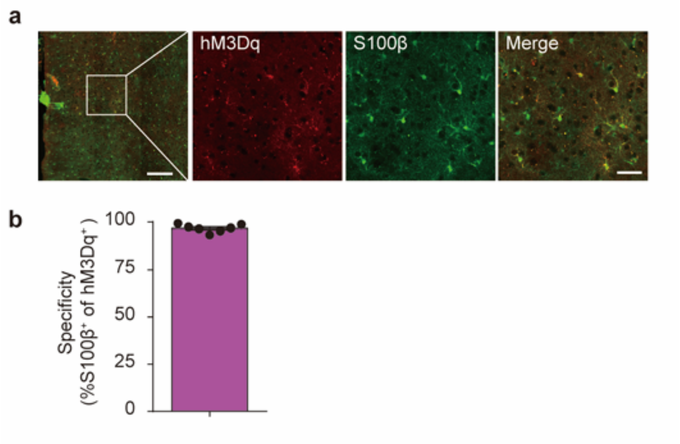

Supplementary Figure 13 The specificity of GFAP::hM3Dq expression.

a, hM3Dq (red) was co-stained with the astrocytic marker S100β (green) in the mPFC. Scale bars: 200 µm, 50 µm. **b**, GFAP::hM3Dq was expressed in mPFC astrocytes with about 95.67% specificity (95.61 ± 0.56%, n =523 cells from 4 mice).

Supplementary Figure 14

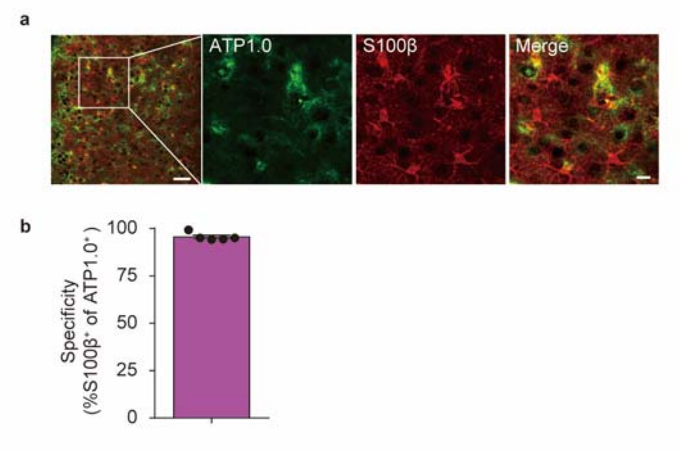

Supplementary Figure 14 The specificity of GfaABC$_1$D::ATP1.0 expression.

a, ATP1.0 (red) was co-stained with the astrocytic marker S100β (green) in the mPFC. Scale bars: 50 µm, 10 µm. **b**, GfaABC$_1$D::ATP1.0 was expressed in mPFC astrocytes with about 95.52% specificity (95.52 ± 0.93%, n = 443 cells from 3 mice).

Supplementary Figure 15

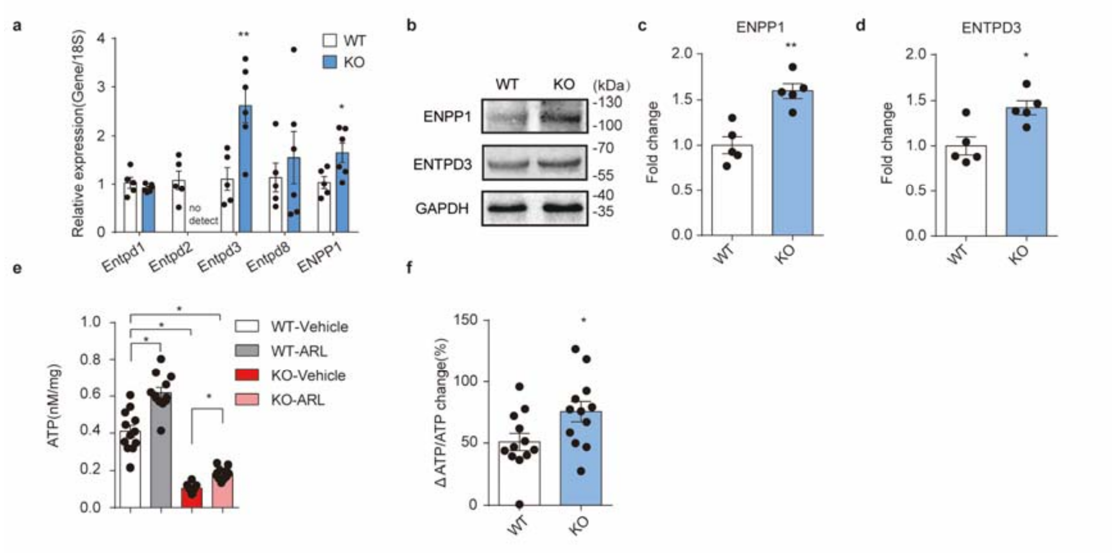

Supplementary Figure 15 Contribution of increased ecto-ATPases expression to the lower level of extracellular ATP in cultured astrocytes from the mPFC of IP3R2 KO mice.

a, The mRNA levels of ecto-ATPases in cultured astrocytes from the mPFC of IP3R2 WT and KO mice. (Entpd1, $U = 9.5$, $P = 0.355$, n =5/6; Entpd2, *not detected in IP3R2 KO mice.* Entpd3, $t_9 = 3.416$, $P = 0.0077$, n =5/6; Entpd8, $t_9 = 0.6310$, $P = 0.5438$, n =5/6; ENPP1, $t_9 = 2.470$, $P = 0.0356$, n =5/6). **b-d**, Western blot showing the protein expression levels of ecto-ATPase ENPP1 and ENTPD3 in IP3R2 WT and KO mice. (**c,** ENPP1: *WT vs.KO*, $t_8 = 4.848$, $P = 0.0013$, $n = 5$; **d,** ENTPD3:*WT vs.KO*, $t_8 = 3.347$, $P = 0.0101$, $n = 5$). **e**, The extracellular ATP levels of cultured astrocytes from IP3R2 WT and KO mice treated with the ATPase inhibitor ARL67156 (50 µM). (one-way ANOVA, F =141.7, $P = 0$, n =12). **f**, ARL treatment produced a bigger increase in ATP change in cultured astrocytes from IP3R2 KO mice compared to IP3R2 WT mice (t= 4.185, $P = 0.0139$). WT, wild-type mice; KO, IP3R2 null mutant

mice. The data are presented as the mean ± SEM. Two-tailed unpaired t test were used (**a**, **c**, **d**, **f**), otherwise Mann-Whitney U test (**a**). One-way ANOVA with Tukey's multiple comparison post hoc test (**e**). *P < 0.05, **P < 0.01. Comparisons with no asterisk had a P > 0.05 and were considered not significant.

Supplementary Figure 16

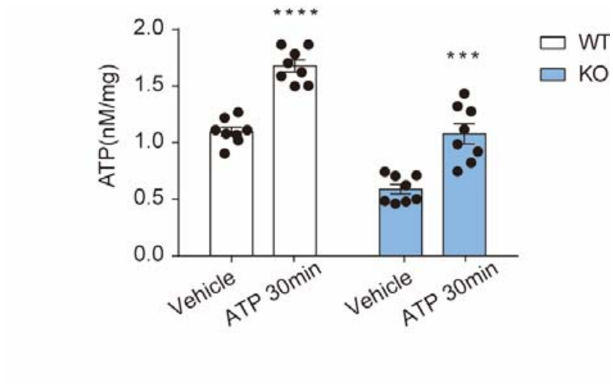

Supplementary Figure 16 The acute injection of ATP increased ATP levels in the mPFC of IP3R2 WT and KO mice. IP3R2 WT and KO mice that were injected with saline (vehicle) or ATP (125 mg kg^{-1}, i.p.) were sacrificed 30 min after injections. The mPFC slices were immediately sectioned and incubated in oxygenized ACSF for 12 min. Measurement of ATP levels indicated that acute treatments with ATP reversed the lower ATP levels in IP3R2 KO mice relative to controls. IP3R2 WT (t_{14} =8.798, P = 0. $9*10^{-5}$, n = 8); IP3R2 KO (t_{14}= 5, P = 0.0002, n = 8). WT, wild-type mice; KO, IP3R2 null mutant mice. The data are presented as the mean ± SEM. Two-tailed unpaired t test was used. ***P<0.001, ****P<0.0001.

Supplementary Figure 17

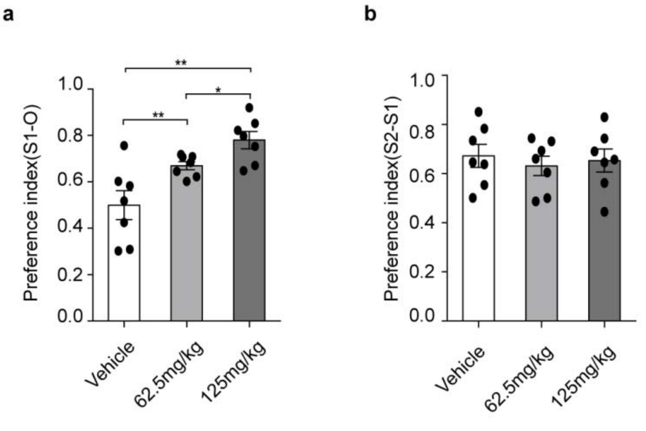

Supplementary Figure 17 ATP treatment improves social interaction in IP3R2 KO mice in the three-chamber test in a dose-dependent manner.

a-b, A single i.p. injection of ATP (62.5 mg/kg or 125 mg/kg) rescued the impaired social interaction in IP3R2 KO mice in the social approach session (**a**: $F_{2,18} = 10.74$, P= 0.0009, n = 7; Vehicle vs 62.5 mg/kg, P =0.00226; Vehicle vs 125mg/kg, P = 0.0023; 62.5mg/kg vs 125mg/kg, P = 0.0189) but not in the social novelty session (**b**: $F_{2,18} = 0.2109$, P = 0.8119, n = 7). The data are presented as the mean ± SEM. *P < 0.05, **P < 0.01. One-way ANOVA with Fisher's LSD multiple comparison post hoc test. Comparisons with no asterisk had a P > 0.05 and were considered not significant.

Supplementary Figure 18

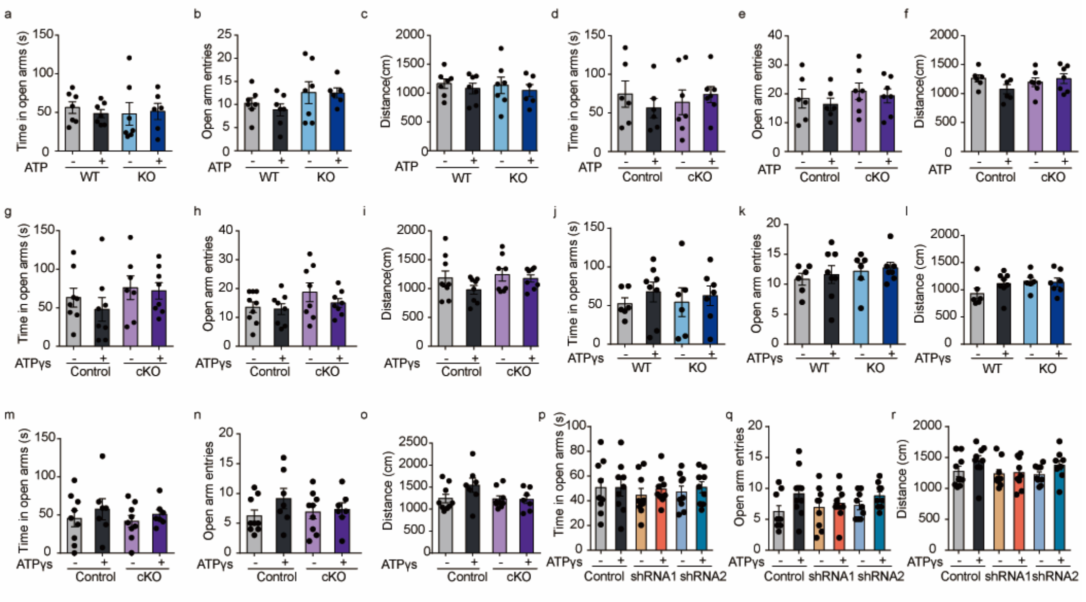

Supplementary Figure 18 IP3R2 mutant and control mice treated with ATP or ATPγS show normal anxiety-like behaviors.

a-c, In the EPM, an i.p. injection of ATP (125 mg/kg) had no effect on the time spent in the open arms (**a**: $F_{3,23}$ = 0.1445, P = 0.9321, n = 7,7,7,6), the number of entries into the open arms (**b**: $F_{3,23}$ =1.283, P =0.3039, n = 7,7,7,6), or the distance traveled (**c**: $F_{3,23}$ = 0.2407, P = 0.8670, n = 7,7,7,6) between WT and IP3R2 KO mice. **d-f**, In the EPM, an i.p. injection of ATP (125 mg/kg) had no effect on the time spent in the open arms (**d**: $F_{3,22}$ = 0.3641, P = 0.7796, n = 6,6,6,7), the number of entries into the open arms (**e**: $F_{3,22}$ = 0.474, P = 0.7063, n = 6,6,6,7), or the distance traveled (**f**: $F_{3,22}$ = 1.194, P = 0.3350, n = 6,6,6,7) between control and IP3R2 cKO mice. **g-i,** In the EPM, ATPγS treatment (50 μM, i.c.v.) had no effect on the time spent in the open arms (**g**: $F_{3,27}$ = 0.8598, P = 0.4738, n = 8,8,7,8), the number of entries into the open arms (**h**: $F_{3,27}$ = 0.7041, P = 0.5579, n = 8,8,7,8) or the distance traveled (**i**: $F_{3,27}$ = 1.209, P =

0.3255, n = 8,8,7,8) between control and IP3R2 cKO mice. **j-l**: In the EPM, ATPγS treatment (50 μM, intra-mPFC) had no effect on the time spent in the open arms (**j**: $F_{3,23}$ = 0.2874, P = 0.8340, n = 6,8,6,7), the number of entries into the open arms (**k**: $F_{3,23}$ = 0.3861, P = 0.0.7640, n = 6,8,6,7), or the distance traveled (**l**: $F_{3,23}$ = 1.504, P = 0.2399, n =6,8,6,7) between WT and IP3R2 KO mice. **m-o**: In the EPM, ATPγS treatment (50 μM, intra-mPFC) had no effect on the time spent in the open arms (**m**: $F_{3,28}$ = 0.4774, P = 0.7005, n = 9,7,9,7), the number of entries into the open arms (**n**: $F_{3,28}$ = 0.7041, P = 0.9790, n = 9,7,9,7), or the distance traveled (**o**: $F_{3,28}$ = 1. 951, P = 0.1443, n = 9,7,9,7) between control and IP3R2 cKO mice. **p-r**: In the EPM, ATPγS treatment (50 μM, intra-mPFC) had no effect on the time spent in the open arms (**p**: F5$_{3,48}$ = 0.2119, P = 0.9558, n = 9), the number of entries into the open arms (**q**: F_5 $_{,48}$ = 1.282, P = 0.2870, n = 9), or the distance traveled (**r**: $F_{5,48}$= 1.355, P = 0.2587, n = 9) between Control and AAV-GFAP-IP3R2 shRNA1 or AAV-GFAP-IP3R2 shRNA2 mice. The data are presented as the mean ± SEM. One-way ANOVA with Tukey's multiple comparison post hoc test. Comparisons with no asterisk had a P > 0.05 and were considered not significant.

Supplementary Figure 19

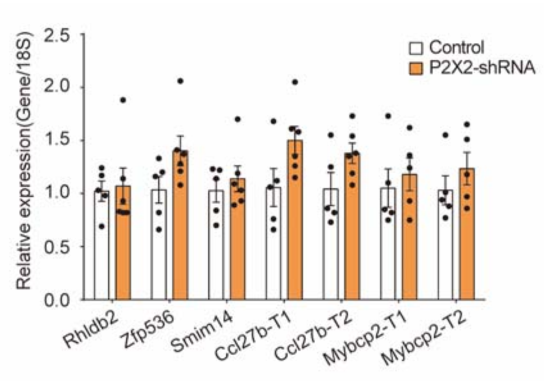

Supplementary Figure 19 No alterations in the mRNA levels of the potential genes that may be targeted by P2X2R-shRNAs in the mPFC of C57BL/6J mice treated with P2X2R-shRNA. The mRNA levels of 7 genes that may be targeted by P2X2R-shRNAs in the mPFC of C57BL/6J mice were not changed after P2X2R-shRNAs treatment (Rhldb2, $t_9 = 0.2324$, $P = 0.8214$, n =5/6; Zgp536, $t_9 =$ 1.911, $P = 0.0884$ n =5/6; Smim14, $t_9 = 0.6805$, $P = 0.5133$, n =5/6; Ccl27b-T1, $t_9 =$ 2.038, $P = 0.072$, n =5/6; Ccl27b-T2, $t_9 = 1.908$, $P = 0.0887$, n =5/6; Mybcp2-T1, t_9 = 0.5453, $P = 0.6004$, n =5/6; Mybcp2-T2, $t_9 = 1.010$, $P = 0.342$, n =5/6). The data are presented as the mean ± SEM. Two-tailed unpaired t test. Comparisons with no asterisk had a P > 0.05 and were considered not significant.

Supplementary Figure 20

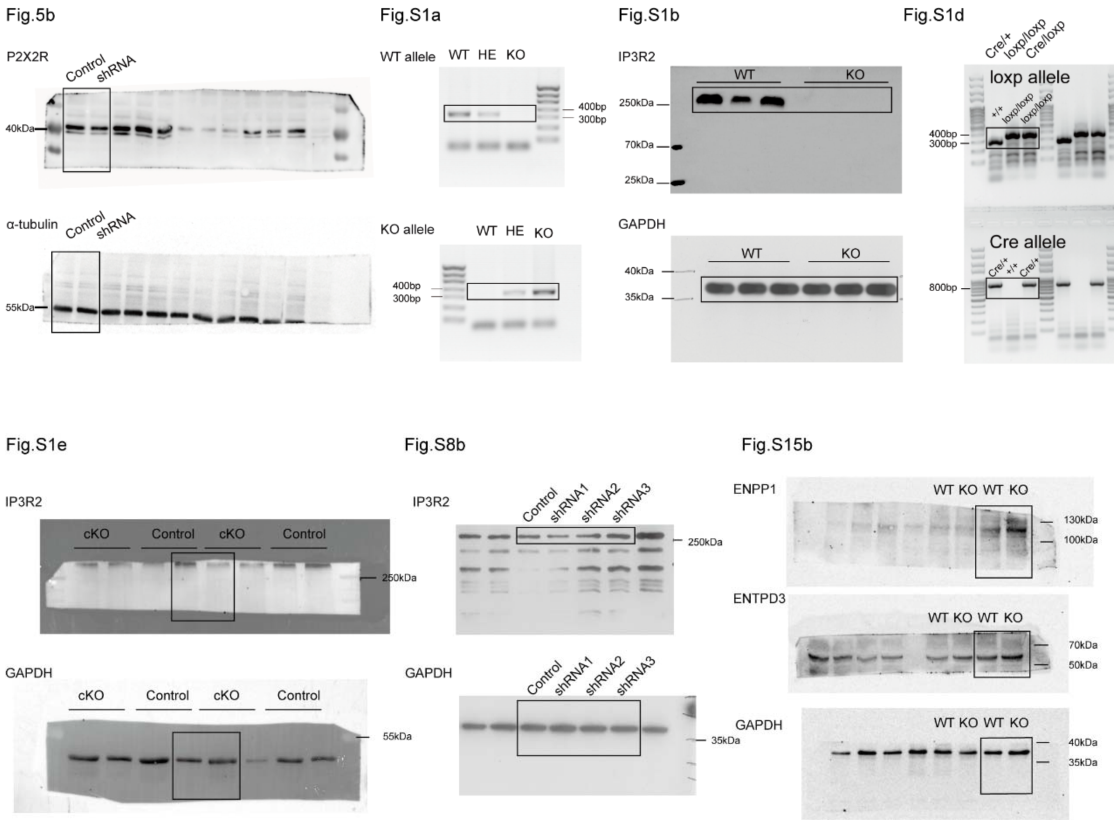

Supplementary Figure 20 Full length images of the blots presented in the figures.
