# Supplementary material for: Impaired calcium signaling in astrocytes modulates autism spectrum disorder-like behaviors in mice
Source: Nat Commun. 2021 May 31;12:3321. doi: 10.1038/s41467-021-23843-0 (PMC8166865; doi:10.1038/s41467-021-23843-0)
Supplement: Supplementary file 7 — Reporting Summary [file 41467_2021_23843_MOESM7_ESM.pdf]

natureresearch

Corresponding author(s): T.M. Gao

Last updated by author(s): Dec 22, 2020

# Reporting Summary

Nature Research wishes to improve the reproducibility of the work that we publish. This form provides structure for consistency and transparency in reporting. For further information on Nature Research policies, see Authors & Referees and the Editorial Policy Checklist .

## Statistics

For all statistical analyses, confirm that the following items are present in the figure legend, table legend, main text, or Methods section.

| n/a | Confirmed | |
|---|---|---|
| ☐ | ☒ | The exact sample size (*n*) for each experimental group/condition, given as a discrete number and unit of measurement |
| ☐ | ☒ | A statement on whether measurements were taken from distinct samples or whether the same sample was measured repeatedly |
| ☐ | ☒ | The statistical test(s) used AND whether they are one- or two-sided<br>*Only common tests should be described solely by name; describe more complex techniques in the Methods section.* |
| ☐ | ☒ | A description of all covariates tested |
| ☐ | ☒ | A description of any assumptions or corrections, such as tests of normality and adjustment for multiple comparisons |
| ☐ | ☒ | A full description of the statistical parameters including central tendency (e.g. means) or other basic estimates (e.g. regression coefficient) AND variation (e.g. standard deviation) or associated estimates of uncertainty (e.g. confidence intervals) |
| ☐ | ☒ | For null hypothesis testing, the test statistic (e.g. *F*, *t*, *r*) with confidence intervals, effect sizes, degrees of freedom and *P* value noted<br>*Give P values as exact values whenever suitable.* |
| ☒ | ☐ | For Bayesian analysis, information on the choice of priors and Markov chain Monte Carlo settings |
| ☐ | ☒ | For hierarchical and complex designs, identification of the appropriate level for tests and full reporting of outcomes |
| ☒ | ☐ | Estimates of effect sizes (e.g. Cohen's *d*, Pearson's *r*), indicating how they were calculated |

*Our web collection on statistics for biologists contains articles on many of the points above.*

## Software and code

Policy information about availability of computer code

| | |
|---|---|
| Data collection | Ethovision XT 11.0 software (Noldus) was used for animal tracking. Image processing was performed using custom routines for the Fiji distribution of ImageJ (Version 1.50i); Multiclamp 700B (Axon Instrument, Molecular Devices) was used for electrophysiological recordings. |
| Data analysis | pClamp 10.2 software (v.10.6.2.2., Molecular Devices) and MiniAnalysis software (v6.03, Synaptosoft Inc. USA) were used to analyzed electrophysiological data. QuantaSoft software (Bio-Rad, v 1.7.4.0917) was used for analysis of droplet digital PCR data. Statistical analyses were performed in SPSS 13.0 software. |

For manuscripts utilizing custom algorithms or software that are central to the research but not yet described in published literature, software must be made available to editors/reviewers. We strongly encourage code deposition in a community repository (e.g. GitHub). See the Nature Research guidelines for submitting code & software for further information.

## Data

Policy information about availability of data

All manuscripts must include a data availability statement. This statement should provide the following information, where applicable:

- Accession codes, unique identifiers, or web links for publicly available datasets
- A list of figures that have associated raw data
- A description of any restrictions on data availability

The data of this study are available from the corresponding author upon reasonable request.



October 2018

# Field-specific reporting

Please select the one below that is the best fit for your research. If you are not sure, read the appropriate sections before making your selection.

- [x] Life sciences
- [ ] Behavioural & social sciences
- [ ] Ecological, evolutionary & environmental sciences

For a reference copy of the document with all sections, see nature.com/documents/nr-reporting-summary-flat.pdf

# Life sciences study design

All studies must disclose on these points even when the disclosure is negative.

| | |
|---|---|
| Sample size | No statistical methods were used to predetermine sample size, but sample sizes are consistent with those generally employed in the field(e.g. Bicks et at, 2020, Sun et al 2016). |
| Data exclusions | No data were excluded from the analysis. |
| Replication | For all studies, independent animals were used as replicates. Replication studies confirmed our results in all experiments. |
| Randomization | Animals were randomly assigned numbers and tested blind for the experimental condition. |
| Blinding | All behavioral experiments were scored by an individual blind to the genotype and experimental design. |

# Reporting for specific materials, systems and methods

We require information from authors about some types of materials, experimental systems and methods used in many studies. Here, indicate whether each material, system or method listed is relevant to your study. If you are not sure if a list item applies to your research, read the appropriate section before selecting a response.

## Materials & experimental systems

| n/a | Involved in the study | |
|---|---|---|
| [ ] | [x] | Antibodies |
| [x] | [ ] | Eukaryotic cell lines |
| [x] | [ ] | Palaeontology |
| [ ] | [x] | Animals and other organisms |
| [x] | [ ] | Human research participants |
| [x] | [ ] | Clinical data |

## Methods

| n/a | Involved in the study | |
|---|---|---|
| [x] | [ ] | ChIP-seq |
| [ ] | [x] | Flow cytometry |
| [x] | [ ] | MRI-based neuroimaging |

## Antibodies

| | |
|---|---|
| Antibodies used | Primary antibodies: polyclonal rabbit anti-IP3R2 antibody (Gifted from Ju Chen's lab,1:500); polyclonal rabbit anti-P2X2 antibody (Abcam, ab48864) 1:1000; monoclonal rat anti-ACSA-2 antibody (Miltenyi Biotec, 130-102-365) 1:20 for FACS; monoclonal rabbit anti-NeuN antibody (Cell Signaling Technology, 24307) 1:500; monoclonal rabbit anti-S100β antibody (Abcam, ab52642) 1:100; Polyclonal rabbit anti-ENTPD3 antibody (Proteintech, 13021-1-AP) 1:1000; Polyclonal rabbit anti-ENPP1 antibody (Cell Signaling Technology, 2061) 1:1000. Secondary antibodies (all from Invitrogen): Alexa Fluor 488 (A11034) 1:500, Alexa Fluor 594 (A11005) 1:500, Alexa Fluor 647(A327733) 1:500;HRP-conjugated secondary antibody (ZB-2305, ZSGB-Bio)1:10000. |
| Validation | All antibodies used were commercial and validated except the polyclonal rabbit anti-IP3R2 antibody, which was a gift from Ju Chen and has been used and cited by over 4 papers; anti-P2X2 antibody, anti-ACSA-2 antibody, anti-NeuN antibody, anti-S100β antibody, anti-ENTPD3 antibody, and anti-ENPP1 antibody haave been used and cited by over 10 papers. The secondary antibodies were also validated and has been used and cited by over 100 papers. |

## Animals and other organisms

Policy information about studies involving animals; ARRIVE guidelines recommended for reporting animal research

| | |
|---|---|
| Laboratory animals | Male mice (8-12 weeks of age) of the following strains were used: Adult C57BL/6J mice (provided by Guangzhou Southern Medical University Animal Center); IP3R2 knock-out mice and IP3R2 floxed mice ( gifted from Dr. Ju Chen); Aldh1L1-CreER mice (generated by Model Animal Research Center of Nanjing University, China); Ai14 reporter line (purchased from the Jackson Laboratory,007914). 1-2-day-old IP3R2 knock-out mice were used for cell culture. Mice were housed in standard laboratory cages (4-5 per cage) at temperature 24 ± 1 degree Celsius, humidity ~60%, maintained on a 12-h light/dark cycle with lights on at 8:00 a.m., and provided food and water ad libitum. |

| | |
|---|---|
| Wild animals | The study did not involve the wild animals. |
| Field-collected samples | The study did not involve samples collected from the field. |
| Ethics oversight | The animal care and use protocol was approved by Animal Care and Use Committee of Southern Medical University and Animal Facility at the Laboratory Animal Center, Southern Medical University,China. |

Note that full information on the approval of the study protocol must also be provided in the manuscript.

# Flow Cytometry

## Plots

Confirm that:

- [x] The axis labels state the marker and fluorochrome used (e.g. CD4-FITC).
- [x] The axis scales are clearly visible. Include numbers along axes only for bottom left plot of group (a 'group' is an analysis of identical markers).
- [x] All plots are contour plots with outliers or pseudocolor plots.
- [x] A numerical value for number of cells or percentage (with statistics) is provided.

## Methodology

| | |
|---|---|
| Sample preparation | *Describe the sample preparation, detailing the biological source of the cells and any tissue processing steps used.* |
| Instrument | Beckman MoFlo XDP Cell Sorter system |
| Software | *Describe the software used to collect and analyze the flow cytometry data. For custom code that has been deposited into a community repository, provide accession details.* |
| Cell population abundance | *Describe the abundance of the relevant cell populations within post-sort fractions, providing details on the purity of the samples and how it was determined.* |
| Gating strategy | *Describe the gating strategy used for all relevant experiments, specifying the preliminary FSC/SSC gates of the starting cell population, indicating where boundaries between "positive" and "negative" staining cell populations are defined.* |

- [ ] Tick this box to confirm that a figure exemplifying the gating strategy is provided in the Supplementary Information.
